# Progress in child nutrition outcomes: insights from India’s recent experience

**DOI:** 10.1186/s12889-025-24436-y

**Published:** 2025-09-30

**Authors:** Ivica Petrikova, Narender Kumar

**Affiliations:** 1 Royal Holloway, University of London, Egham, UK; 2 Jawaharlal Nehru University, New Delhi, India

**Keywords:** Child nutrition, India, NFHS, Pandemic, WASH, Animal-sourced food, Rajasthan, Himachal Pradesh

## Abstract

**Background:**

Improvements in child nutrition outcomes have lagged behind India’s recent rapid economic growth, a phenomenon known as the ‘Indian enigma.’ Between 2015–16 and 2019–21, stunting and wasting rates declined only slightly, with some Indian states even experiencing worsening malnutrition. This study investigates the factors driving these trends, focusing on the impact of the COVID-19 pandemic, children’s dietary quality, access to water, sanitation, and hygiene (WASH), and the role of government nutrition programmes.

**Methods:**

The study employs a mixed-methods approach, combining a quantitative analysis of data from India’s National Family Health Surveys (NFHS) IV (2015–16) and V (2019–21) with a qualitative comparative case study of Rajasthan and Himachal Pradesh—two states with contrasting malnutrition trends. Individual- and district-level regression models were used to assess the effects of dietary diversity, WASH access, and government programmes, whilst interviews with policy makers in Rajasthan and Himachal have provided insights into programme implementation and local responses during the pandemic.

**Results:**

Our findings confirm that dietary diversity, particularly consumption of animal-sourced foods, and improved WASH access are key drivers of better nutrition outcomes in India. However, contrary to expectations, we do not find a consistently negative relationship between the COVID-19 pandemic and child malnutrition. Instead, flexible expansion of some of the welfare programmes during the pandemic along with reductions in children’s disease rates and improvement in some sanitation practices may have mitigated the expected deterioration. Findings from the comparative study of Rajasthan and Himachal further highlight the importance of tailoring welfare programmes to specific local conditions, such as the large proportion of migrant labourers in Himachal.

**Conclusions:**

Our study challenges assumptions about the pandemic’s uniformly negative effects on child nutrition and highlights the importance of resilient, locally tailored safety nets. The findings underscore the need for policy interventions that enhance dietary diversity, sustain WASH improvements, and strengthen the adaptability of food and nutrition programmes to crises.

**Supplementary Information:**

The online version contains supplementary material available at 10.1186/s12889-025-24436-y.

## Background

The Indian economy has grown rapidly for the past three decades, with the highest growth in the world in 2023. However, India’s improvement in children’s nutrition outcomes, widely used as an indicator of countries’ food and nutrition security, has not been commensurate with the fast economic growth, a paradox referred to as the ‘Indian enigma’ [1]. Between 2015–16 and 2019–21, key malnutrition indicators including child stunting (too short for age) and wasting (too thin for height) declined but only marginally—from 38 to 36 percent and from 21 to 19 percent [2, 3]. This slight reduction additionally hides a lot of variation, with child stunting and wasting actually increasing in one third of Indian states in that time. Given the negative health, educational, and economic implications of child malnutrition for the individuals affected, their families, communities, and countries’ development [4], further investigation of India’s persistently high rates of child malnutrition is imperative.

Using data from NFHS 4 (2015–16) [2] and NFHS 5 (2019–21) [3], alongside a comparative case study of two Indian states, Rajasthan and Himachal Pradesh, this article investigates the factors underlying India’s limited progress in reducing child malnutrition. We focus particularly on the impact of the Covid-19 pandemic and its interaction with children’s dietary patterns, access to sanitation, and the performance of key relevant government programmes. As expected, we find that diverse diets, especially those including animal-sourced foods (ASFs), and improved sanitation are associated with better nutrition outcomes. However, our findings challenge our original assumption that the pandemic had a uniformly negative effect. Contrary to expectations, we do not observe a deterioration in children’s nutrition outcomes during the early part of the pandemic. Our analysis instead suggests that reduced disease incidence and improvements in sanitation, potentially along with the flexible expansion of welfare programmes, helped mitigate the pandemic’s adverse nutritional effects.

The article’s key contributions to existing literature are hence three-fold. First, the article sheds new light on the ‘Indian enigma,’ by examining the latest available data on Indian children’s nutrition outcomes and their drivers. Second, unlike most research on child nutrition, it supplements quantitative analysis with qualitative, enhancing the robustness, validity, and contextual depth of its findings. Finally, it contributes to the growing body of pandemic-related research by showcasing an example of Covid-19’s nutritional impact being at least partially buffered by changes in people’s health-related behaviour and the government’s response.

The article proceeds in the following manner. The next section reviews existing literature on the drivers of children’s nutrition outcomes generally and specifically in India. We then describe our data, and the methods utilised to analyse them, and present our results. The last section discusses the relevance of our findings and their implications for policy and future research.

### Drivers of child nutrition outcomes

Two factors have been highlighted as particularly crucial for children’s nutrition outcomes in India: the quality of 1. children’s diets and of 2. water, sanitation, and hygiene (WASH) access. These factors are especially important in children’s first 1000 days of life, when growth and health trajectories are most sensitive. The Indian government has attempted to address these issues through a range of government programmes for decades, with varying success. Both the issues and the programmes in place to address them were significantly affected by the Covid-19 pandemic, with notable consequences for children’s nutrition outcomes.

Regarding children’s diets, the World Health Organisation (WHO) recommends exclusive breastfeeding for the first six months, followed by a timely introduction of diverse complementary foods, a so-called ‘minimum acceptable diet’ (MAD) [5]. Research consistently links timely weaning and growth [6–9] as well as higher dietary diversity/MAD and growth [10–13]. In the Indian context specifically, low consumption of iron-rich and animal-sourced foods remains a critical barrier to improved children’s nutrition [7]. Livestock ownership could facilitate the consumption of animal-sourced foods, given the generally high market price of these items, but the relationship is complex since livestock ownership might in addition to greater consumption of animal-sourced foods also increase the incidence of enteric diseases [13–15].

Poor water, sanitation, and hygiene services are another key factor driving children’s malnourishment across low- and middle-income countries generally and in India specifically. Inadequate WASH increases the likelihood of diarrhoeal diseases and diarrhoea has been one of the leading causes of malnutrition in children under five years old [5, 16–18]. India has historically had very high levels of open defecation and the inter-district differences in open-defecation rates were found to explain up to 55 percent of variation in their child stunting rates in the 2010s [19]. Gastro-intestinal infections caused by poor-quality drinking water and higher rates of malaria brought about by greater water pooling in areas with deficient sanitation have been described as additional pathways between poor WASH and high child malnutrition rates [20].

### India’s nutrition-related government programmes

India’s approach to food and nutrition security has been since 2013 framed through the National Food Security Act (NFSA), which has legally enshrined Indian people’s right to food [21]. The NFSA is based primarily on three social programmes: the Public Distribution System (PDS), the Integrated Child Development Scheme (ICDS), and the Mid-Day Meal Scheme (MDMS). Water, sanitation, and hygiene programmes are also of interest here.

The PDS was established after World War Two with the two-pronged aim of bolstering national food production and improving people’s access to food and to date it has remained India’s largest national food-access programme [22]. It has always involved the sale of highly subsidised rice and wheat to beneficiaries but has varied in coverage, oscillating between universal and more narrowly targeted. Since the implementation of the NFSA, the PDS has covered 75 percent of rural and 50 percent of urban population (the less wealthy sections), whom it grants the right to purchase 5 kg of subsidised grains, mostly wheat and rice, per month [23].^1^

The ICDS and MDMS are aimed at addressing food and nutrition insecurity amongst particularly vulnerable populations. The ICDS was established in 1975 with a focus on encouraging correct nutritional, feeding, hygiene, and health practices in pregnant and lactating women and in children under six years of age [24]. Specific interventions within the scheme have included the provision of supplementary nutrition to young children and their mothers, of nutritional and health education to mothers, and of growth-monitoring, de-worming, and pre-school education to children [22]. The services are provided through an extensive national network of *anganwadi* centres (AWCs). Meanwhile, the MDMS has been implemented nationally since 2001, to improve nutrition along with school attendance amongst primary-school pupils [25].^2^

Finally, given that deficient water and sanitation access have been identified as key drivers of India’s high malnutrition rates [19], successive Indian governments implemented drives to build toilets and eradicate open defecation. The most recent and arguably most successful such programme to date has been the Swachh Bharat, implemented from 2014 to 2019 [26, 27]. The programme has claimed to have eradicated open defecation; whilst survey data do not support that conclusion, they do indicate that a significant reduction in open defecation has taken place [3].

Evidence on the effects of these government programmes on food and nutrition security has been mixed. Some studies found the PDS to have increased caloric consumption [28–30], whilst others did not [31, 32]. The programme’s positive effect on people’s access to food arguably increased after the reform under the NFSA [30, 33], but its impact on nutrition outcomes is more questionable, with beneficiaries found to consume more wheat and rice at the expense of nutritious grains, fruit, and dairy [31, 34, 35]. The ICDS has been more successful in improving nutrition outcomes. Whilst earlier studies of the programme showed minimal impact [24, 36], some more recent studies identified a positive effect for some groups of children [37–39]. The Swachh Bharat programme reduced open defecation rates, the occurrence of diarrhoeal diseases, and infant and child mortality [40, 41], although infrastructure issues for waste disposal and other barriers remain [42, 43].

### Covid-19- pandemic and child nutrition in India

Food and nutrition security in India and beyond has been significantly affected by the Covid-19 pandemic and its related impact. The long-term effects of the pandemic in India have not been explored in detail yet; existing literature proposes that the effects have been largely negative due to harmful economic consequences but partly offset by the emergency expansion of the welfare programmes discussed. As many other countries, India implemented a national lockdown in March 2020, which was gradually relaxed two months later. During the second wave of the pandemic in India, in the spring of 2021, some states re-instated a lockdown for several months. Indian schools were, however, closed for longer, for 82 weeks between March 2020 and October 2021 [44]—the longest closure in the world after Uganda.

The economic lockdowns led to complete or partial losses of income in many Indian households, with low-income urban households particularly negatively affected. Food programmes, especially the PDS, were expanded in response. Between March 2020 and November 2022, the Government of India implemented the Pradhan Mantri Garib Kalyan Anna Yojana (PMGKAY), which granted all PDS-eligible households extra 5 kgs of rice or wheat per person and 1 kg of dal per household per month. New beneficiaries were also added to the lists of PDS eligible recipients at the beginning of the pandemic [45]. The ICDS and MDMS were significantly disrupted by the pandemic, however. The MDMS could not serve hot cooked meals to pupils whilst schools were closed and the need for social distancing also led to temporary closures of some ICDS Anganwadi centres. The programmes attempted to swiftly shift to the provision of dry-food rations and cash transfers but with mixed results.

Two studies, one from Hyderabad [46] and one from Bihar [47], described how the pandemic significantly increased household food insecurity. Another study [48] explored the effects of the pandemic on children’s weight-for-age scores in selected districts in Bihar, Uttar Pradesh, and Odisha and found the effect to be also significantly negative, although not for all children. One of the pathways through which the deterioration is believed to have occurred was via decline in dietary quality in response to greater economic deprivation [48, 49]. The effects of the pandemic on the other main pathway, sanitation, have been more mixed, with some increase in open defecation but improvement in hygiene practices [43, 50]. Other relevant studies [51–54] noted the disruption of welfare programmes during the pandemic but also some positive trends—for example, that the lower social contact between households during the pandemic led to fewer diseases amongst children [55] and public-service delivery innovations such as combining Vitamin A-supplementation with immunisations and using WhatsApp groups to share information about child feeding [51].

### Research focus

Building on this context, in this article we investigate the factors underlying India’s slow progress in reducing child malnutrition, with specific attention paid to: 1. the effect of the Covid-19 pandemic—we expect the pandemic to have undermined gains made in reducing child malnutrition rates but less so in households and areas where welfare programmes proved to be resilient and/or were expanded (H1); 2. the role of children’s dietary quality—we expect that primarily households and areas with greater improvement in children’s diets (MAD, ASF consumption) experienced greater reduction in child malnutrition rates; however, we expect the pandemic to have worsened dietary trends (H2); and 3. the role of WASH—we expect households and areas with greater WASH access to have experienced more reduction in child malnutrition rates, with the pandemic accelerating improvements in some aspects (H3).

## Methods

**Fig. 1:**
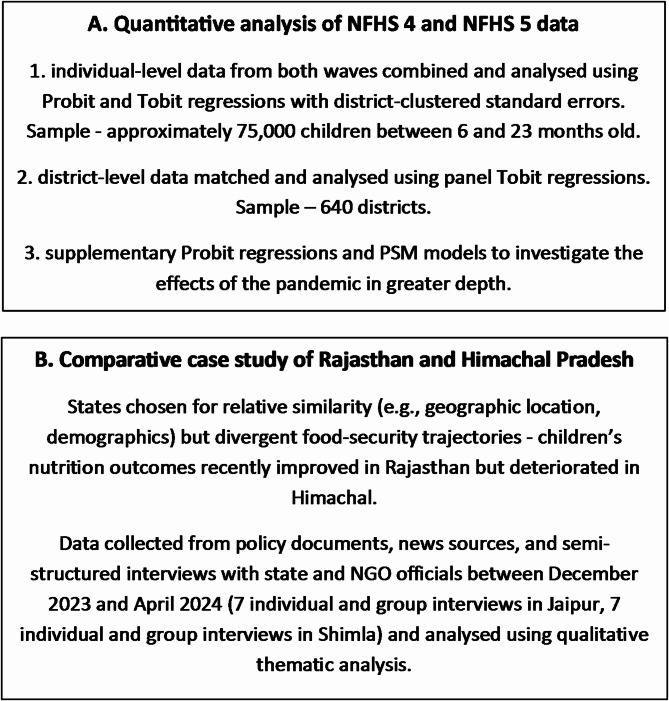
Article’s analytical framework summary. *Source*: authors’ own work

This study uses mixed research methods. The first part is a quantitative analysis of data from NFHS4 [2] and NFHS5 [3]. Individual-level data from the two survey waves are first analysed jointly; a panel-data analysis of matched district-level data follows. The second part of the study involves a qualitative comparative analysis of two Indian states–Rajasthan and Himachal Pradesh. The approach is graphically summarised in Fig. 1 below.

### Quantitative national analysis

First, the study analyses data from more than 75,000 Indian children between 6 and 23 months old gathered by NFHS4 (~ 40,000) and NFHS5 (~ 35,000). The National Family Health Survey is a nationally representative, India-wide household survey that collects data on health-related and socio-economic measures. The first wave of the survey was rolled out in 1992, as part of the United States Agency for International Development’s (USAID) Demographic and Health Survey (DHS) programme, which encompassed more than 90 countries. NFHS uses two-stage stratified cluster sampling design; the fourth wave in India covered overall approximately 600,000 and the fifth wave 640,000 households.

We chose the lower cut-off age of 6 months for our sample due to the WHO recommendation for children from that age onwards to be ‘weaned’; i.e. to receive semisolid food as a supplement to breast milk [5]. The upper cut-off point was chosen because the first two years of children’s lives are considered the most crucial to ensuring good nutrition and health outcomes in later life [56] and because data on children’s diets were collected only up to that age. We first analyse individual-level data combined from the two survey waves^3^ and second averaged and matched district-level data. Most district boundaries remained unchanged between 2015–16 and 2019; where change did occur, it was usually through subdivision into smaller sections. We therefore work with the 640 2015–16 districts, to which we matched the 2019–21 districts. In estimating the effects of the Covid-19 pandemic, we exploit the fact that one-third of NFHS V survey data collection occurred after the first wave of Covid-19 in India (November 2020–May 2021).

Our individual-level empirical models examine children’s nutrition outcomes as a function of children’s diets and other characteristics as well as their mothers’, households’, and communal characteristics. The district-level models analogously examine the district-level prevalence of children’s nutrition outcomes as a function of district-averaged children’s, their mothers’, and households’ characteristics, under the assumption that the surveyed households are collectively representative of the districts in which they reside. We estimate the individual-level models using Probit and Tobit regressions that control for the region of residence and whose robust standard errors are clustered by districts [following e.g., 7, 57, 58]. The district-level data are analysed using panel Tobit regressions that control for region. To investigate the effects of the pandemic in greater depth, the basic models are supplemented with further Probit regressions with interactions and Propensity Score Matching (PSM) models, explained later in more detail.^4^

### Qualitative analysis

The second part of the analysis comprises a comparative case study of Rajasthan and Himachal Pradesh. These two northern Indian states were selected due to their geographical proximity and relative demographic similarities, in terms of their ethnic composition and socio-economic characteristics, but their divergence in recent food and nutrition-security trajectories. Whilst child malnutrition measures in Rajasthan improved significantly between NFHS4 and NFHS5, the opposite happened in Himachal.^5^

Data relevant to the two states were collected from policy documents, news sources, and semi-structured online and in-person interviews with a range of state officials between December 2023 and April 2024 (7 individual and group interviews in Jaipur, 7 individual and group interviews in Shimla). These data were analysed using thematic analysis.

### Variables

#### Dependent variables

The study examines four malnutrition measures in children—stunting (too short for age), underweight (too light for age), wasting (too light for height), and anaemia (lower than normal red-blood-cell count). All four types of malnutrition can be brought about by deficient feeding, but stunting is generally reflective of longer-term whilst wasting of shorter-term nutrition deprivation. Underweight can be a result of either stunting or wasting whereas anaemia may be caused by iron-deficient diets, alongside frequent diarrhoea and/or intestinal parasites [58, 59]. The study further investigates three other nutrition outcomes—height-for-age (Z scores), weight-for-age (Z scores), and weight-for-height (Z scores).^6^

#### Key independent variables

The main variables of interest in this study involve the pandemic, government’s welfare programmes, children’s diets, WASH, and their interaction. The pandemic variable relates only to NFHS 5 data and takes on a value of 1 when the household was surveyed after the first Covid-19 wave in India—i.e., between November 2020 and May 2021. The only relevant programme on which data in the NFHS are collected is the ICDS—we use several relevant variables here, looking at weekly receipts of food, receiving support in pregnancy, and when breastfeeding. We also look at the PDS, MDMS, and Swachh Bharat in the qualitative case study. In terms of child feeding, the variables of interest include whether a child received the WHO-defined ‘minimum acceptable diet’ (MAD), of satisfactory frequency and diversity of food groups, in the 24 h prior to the administration of the survey and whether s/he has received any animal-sourced food (ASF) other than milk. At the individual level, all these are binary variables. We further look at the household ownership of cattle, poultry, and agricultural land (also all binary variables), which might be linked with the children’s diet composition. For WASH, we utilise several variables—whether a household has a private improved sanitation facility (flushing toilet or an improved latrine), whether a household has access to an improved water source (both binary variables), and the prevalence of private improved toilets at the district level. We also look at the availability of water and of soap/ash at handwashing sites (binary variables as well).

#### Other independent variables

Factors other than poor dietary trends and lack of access to WASH linked with a higher likelihood of childhood malnutrition include, at the child level, being born male, preterm, with a low birthweight, later birth order, and a shorter birth interval with a preceding sibling [6, 7, 26, 60, 61]. At the household level, poorer, younger, worse nourished, less educated mothers with more children have also generally been found to be more likely to have malnourished children [6, 20, 27, 62–64]. Other factors identified as consequential in India have included household caste [7, 12], religion [65], and urban versus rural residence [66].

Accordingly, at the child level, we consider whether a child was born prematurely, his/her gender, age, birth order, birth interval with preceding sibling, whether s/he has had diarrhoea and fever in the last two weeks, and whether s/he has been breastfed for six months. At the mother’s level, we control for her age at giving birth, her education level, and whether she is underweight and/or anaemic. From household variables, we include their size, whether they are female headed, how many children under five they have, their caste (scheduled caste, scheduled tribe, other backward caste, or upper caste), and their religion (Hindu, Muslim or other). Finally, we consider if their residence is in a rural or an urban area, whether it is in a coastal district, and its geographical region in India.^7^

## Results

### Summary statistics of NFHS data used

Table 1 displays the summary statistics of all the variables used in the analysis, separately for the two NFHS rounds examined. The individual-level data used are limited to children between 6 and 23 months old, as dietary data are available only for that age group. The dependent variables section demonstrates that within the sample investigated, as is true of the broader sample of children under five years old, stunting, underweight, and wasting rates all declined whilst the rate of anaemia increased between NFHS 4 and 5. Children’s average height-for-age, weight-for-age, and weight-for-height correspondingly increased as well albeit still remaining firmly below the WHO median.

**Table 1:** Descriptive statistics for all the variables used in the analysis. *Source*: authors’ own analysis of NFHS data

	NFHS 4					NFHS 5				
	Obs	Mean	Std. Dev.	Min	Max	Obs	Mean	Std. Dev.	Min	Max
Dependent variables										
Stunted	39,315	0.38	0.48	0	1	36,006	0.36	0.48	0	1
Underweight	39,315	0.34	0.47	0	1	35,827	0.30	0.46	0	1
Wasted	39,315	0.24	0.43	0	1	34,858	0.21	0.41	0	1
Anaemic	39,315	0.59	0.49	0	1	34,477	0.79	0.41	0	1
ZHA	39,315	− 1.40	1.79	− 6.00	5.97	36,006	− 1.23	2.03	− 6.00	6.00
ZWA	39,315	− 1.49	1.25	− 5.94	4.96	35,827	− 1.32	1.36	− 5.98	4.99
ZWH	39,315	− 1.03	1.44	− 5.00	5.00	34,858	− 0.80	1.56	− 5.00	4.96
Main independent variables										
Pandemic	39,315	0.00	0.00	0	0	36,006	0.33	0.47	0	1
Weekly from the ICDS	39,315	0.50	0.50	0	1	36,006	0.49	0.50	0	1
ICDS pregnancy assistance	39,315	0.81	0.39	0	1	36,006	0.87	0.34	0	1
ICDS breastfeeding assistance	39,315	0.74	0.44	0	1	36,006	0.83	0.38	0	1
Minimum acceptable diet	39,315	0.08	0.28	0	1	36,006	0.10	0.30	0	1
Any ASF	39,315	0.35	0.48	0	1	36,006	0.39	0.49	0	1
Any agricultural land	39,315	0.48	0.50	0	1	36,006	0.50	0.50	0	1
Cattle	39,315	0.45	0.50	0	1	36,006	0.45	0.50	0	1
Poultry	39,315	0.19	0.39	0	1	36,006	0.18	0.38	0	1
Private improved toilet	39,315	0.38	0.49	0	1	36,006	0.65	0.48	0	1
Improved water	39,315	0.91	0.29	0	1	36,006	0.92	0.27	0	1
Water at handwashing place	39,315	0.82	0.38	0	1	36,006	0.91	0.28	0	1
Soap or ash at handwashing place	39,315	0.71	0.45	0	1	36,006	0.81	0.39	0	1
District - private improved toilets	39,315	0.40	0.22	0.04	1	36,006	0.64	0.16	0.23	1
Other independent variables										
Male child	39,315	0.52	0.50	0	1	36,006	0.51	0.50	0	1
Child’s age bracket (months)										
6–11	13,030	0.33				11,754	0.33			
12–17	13,125	0.33				12,576	0.35			
18–23	13,160	0.33				11,676	0.32			
Preterm	39,315	0.06	0.24	0	1	36,006	0.12	0.32	0	1
Diarrhoea last 2 weeks	39,315	0.15	0.36	0	1	36,006	0.10	0.31	0	1
Fever last 2 weeks	39,315	0.18	0.38	0	1	36,006	0.16	0.37	0	1
Breastfed until 6 months	39,315	0.87	0.34	0	1	36,006	0.86	0.34	0	1
Birth order	39,315	2.24	1.41	1	15	36,006	2.11	1.29	1	16
Birth interval	39,315	24.18	25.59	0	275	36,006	24.65	27.52	0	264
Mother age at birth	39,315	25.16	4.73	14	49	36,006	25.41	4.69	14	48
Education level										
None/incomplete primary	11,017	0.28				6905	0.19			
Primary	5853	0.15				4231	0.12			
Secondary	19,241	0.49				19,650	0.55			
Tertiary	3204	0.08				5220	0.15			
Mother underweight	39,315	0.30	0.46	0	1	36,006	0.23	0.42	0	1
Mother anaemic	39,315	0.60	0.49	0	1	36,006	0.61	0.49	0	1
HH size	39,315	6.44	2.79	2	41	36,006	6.26	2.61	2	31
No children under 5	39,315	1.82	0.87	0	9	36,006	1.74	0.84	0	8
Female-headed HH	39,315	0.12	0.33	0	1	36,006	0.15	0.36	0	1
Wealth quintile	39,315	2.80	1.39	1	5	36,006	3.12	1.36	1	5
Caste										
UC	6283	0.16				6091	0.17			
SC	8504	0.22				7951	0.22			
ST	8340	0.21				7143	0.20			
OBC	16,188	0.41				14,821	0.41			
Religion										
Hindu	30,687	0.78				28,482	0.79			
Muslim	4,262	0.11				3917	0.11			
Other	4366	0.11				3,607	0.10			
Urban	39,315	0.18	0.39	0	1	36,006	0.17	0.38	0	1
Coastal	39,315	0.07	0.26	0	1	36,006	0.08	0.27	0	1
Region										
Southern	4330	0.11				4667	0.13			
Northeastern	4401	0.11				3916	0.11			
Eastern	9839	0.25				7639	0.21			
Northern	5353	0.14				5425	0.15			
Central	12,759	0.32				10,729	0.30			
Western	2633	0.07				3630	0.10			

Turning to information on the operation of the ICDS, the proportion of households receiving food assistance from the programme at least once a week remained largely flat. The support women received in pregnancy and when breastfeeding rose, however, from 81 and 74 percent in 2015–16 to 87 and 83 percent in 2019–21. Looking at the dietary variables, the percentage of children eating the minimum acceptable diet increased from 8 to 10 percent; similarly, 39 percent of 6–23-month-olds in 2019–21 were reported to have eaten some ASF other than milk in the preceding 24 h compared to 35 percent in 2015–16. The ownership of cattle remained approximately the same throughout (45 percent households), with poultry ownership slightly decreasing (from 19 percent households in 2015–16 to 18 percent in 2019–21) and agricultural land ownership slightly increasing (from 48 to 50 percent households).

Regarding WASH, there was a significant improvement in access to improved sanitation facilities, reflecting successes of the Swachh Bharat, with the proportion of households with private improved toilet rising from 38 percent in 2015–16 to 65 percent in 2019–21. The result is, however, still quite far off the 100 percent target touted by the programme as having been achieved. Household access to an improved water source remained broadly unchanged but the proportion of households with running water and soap/ash at handwashing place grew significantly (81–92 percent for water, 71–81 percent for soap/ash).

Many of the control variables utilised, displayed in the last section of Table 1, also remained broadly similar between NFHS 4 and 5. Mothers’ average age at birth, the children’s gender and age, birth order and interval with preceding sibling, whether the child was breastfed for six months, households’ average size, caste and religious breakdown, and the proportion of households living in urban and coastal areas changed only marginally. Other socio-demographic characteristics changed more notably. In the five years between the surveys, mothers became more educated and less undernourished and households wealthier. The proportion of preterm births increased from 6 to 12 percent whilst the proportion of children who had suffered from diarrhoea or fever in the last two weeks declined from 15 and 18 percent to 10 and 16 percent. Female-headed households also increased in prevalence, from 12 to 15 percent.

### Quantitative results

Tables 2 and 3 below show regression results on key independent variables (Tables A1 and A2 in the online Appendix contain results on the control variables). Table 2 displays results from the analysis of individual-level data whilst Table 3 from the analysis of district-level data. Both sets of results highlight the importance of dietary behaviour and WASH in improving children’s nutrition outcomes. They also suggest that the pandemic’s first wave may have had a less negative effect on India’s child malnutrition rates than expected.^8^

**Table 2:** NFHS4 and 5 individual-level results—key independent variables

	Stunted				Underweight				Wasted				Anaemic				ZHA		ZWA		ZWH	
	(1)		(2)		(3)		(4)		(5)		(6)		(7)		(8)		(9)		(10)		(11)	
NFHS V	0.14	***	0.15	***	0.14	***	0.15	***	0.05		0.05		1.70	***	1.71	***	1.93		− 3.07		8.72	***
	0.03		0.03		0.03		0.03		0.04		0.04		0.05		0.06		2.70		2.01		2.45	
Pandemic	− 0.04		− 0.04		− 0.02		− 0.02		− 0.16	***	− 0.15	***	− 0.29	***	− 0.30	***	− 8.31	*	− 0.12		2.22	
	0.04		0.04		0.04		0.04		0.04		0.04		0.06		0.06		3.37		2.49		2.78	
Weekly food from ICDS	0.01		0.00		0.03		0.02		0.06	**	0.05	*	0.02		0.01		2.22		− 2.24		− 3.61	**
	0.02		0.02		0.02		0.02		0.02		0.02		0.02		0.02		1.65		1.16		1.35	
Minimum acceptable diet	0.03				− 0.03				− 0.01				0.03				− 2.53		0.16		0.03	
	0.03				0.03				0.04				0.04				2.70		1.84		2.19	
Any ASF last 24 h	− 0.08	***			− 0.08	***			− 0.03				− 0.06	*			7.95	***	5.54	***	1.95	
	0.02				0.02				0.02				0.02				1.75		1.13		1.00	
Agricultural land			− 0.05	*			− 0.08	***			− 0.05				− 0.01		6.45	***	5.52	***	1.43	*
			0.02				0.02				0.02				0.02		1.72		1.18		1.38	
Cattle			− 0.02				− 0.02				0.02				0.00		2.67		1.16		1.64	
			0.02				0.02				0.02				0.02		1.69		1.20		1.37	
Poultry			0.02				0.08	**			0.07	*			0.05		− 0.77		− 6.04	***	− 6.29	***
			0.02				0.03				0.03				0.03		2.17		1.60		1.89	
Private improved toilet	− 0.11	***	− 0.11	***	− 0.11	***	− 0.10	***	− 0.07	**	− 0.06	**	− 0.08	**	− 0.07	**	7.51	***	6.63	***	4.72	**
	0.02		0.02		0.02		0.02		0.02		0.02		0.02		0.03		1.71		1.14		1.45	
Improved water source	0.05		0.05		− 0.03		− 0.03		− 0.11	**	− 0.11	**	− 0.01		0.00		− 5.03		3.41		8.72	***
	0.03		0.03		0.03		0.03		0.03		0.03		0.04		0.04		2.89		1.99		2.39	
Water at handwashing place			− 0.02				− 0.07	**			− 0.08	**			0.00		2.95		5.13	***	5.62	**
			0.03				0.03				0.03				0.03		2.31		1.46		1.93	
Soap or ash at handwashing place			− 0.01				− 0.03				0.00				− 0.03							
			0.02				0.02				0.03				0.03							
District − private improved toilets	− 0.20	**	− 0.23	**	− 0.55	***	− 0.59	***	− 0.30	**	− 0.31	**	− 0.05		− 0.06		25.38	***	36.32	***	33.31	***
	0.08		0.08		0.08		0.08		0.09		0.09		0.09		0.09		6.43		5.44		6.56	
*VIF*	1.70		1.71		1.70		1.71		1.70		1.71		1.70		1.71		1.72		1.72		1.72	
*N*	75,321		73,118		76,427		74,196		75,028		72,849		77,695		75,404		73,118		74,196		72,849	

The table lists first coefficients and below standard errors for each key independent variable. ***p < 0.001, **p < 0.01, *p < 0.05. Results for control variables are included in Table A1in the online Appendix

**Table 3:** District− level results from merged NFHS 4 and 5 data–key independent variables

	Stunted		Underweight		Wasted		Anaemic		ZHA		ZWA		ZWH	
	(1)		(2)		(3)		(4)		(5)		(6)		(7)	
NFHS V	0.03	***	0.04	***	0.01		0.16	***	− 57.30	***	− 61.75	***	− 58.59	***
	0.00		0.00		0.01		0.01		4.62		4.40		3.75	
Pandemic	− 0.02	***	− 0.03	***	− 0.03	***	− 0.06	***	0.78		7.28		7.54	
	0.01		0.01		0.01		0.01		5.59		5.32		4.52	
Weekly food from ICDS	0.01		0.01		0.01		0.03		− 11.18		− 11.42		− 8.14	
	0.01		0.01		0.01		0.02		9.09		8.65		7.36	
Minimum acceptable diet	0.03		− 0.03		0.00		− 0.11		− 29.30		− 1.27		− 6.33	
	0.03		0.03		0.03		0.06		33.77		32.13		27.37	
Any ASF	− 0.07	***	− 0.09	***	− 0.07	***	− 0.07	*	53.28	**	51.50	**	57.52	***
	0.02		0.02		0.02		0.04		18.72		17.81		15.18	
Agricultural land	0.02		− 0.02		− 0.01		− 0.03		3.44		11.59		13.21	
	0.02		0.02		0.01		0.03		14.14		13.45		11.46	
Cattle	− 0.03		− 0.01		0.05	**	0.13	***	20.56		8.70		13.59	
	0.02		0.02		0.02		0.03		17.49		16.64		14.19	
Poultry	0.00		0.07	***	0.05	**	0.03		3.41		− 17.33		− 23.97	
	0.02		0.02		0.02		0.03		15.39		14.65		12.47	
Private improved toilets	− 0.06	***	− 0.04	*	0.02		− 0.07	**	16.10		2.51		3.71	
	0.02		0.02		0.02		0.03		14.88		14.16		12.07	
Improved water	0.06	**	0.03		− 0.04	*	0.04		− 18.86		− 0.38		− 1.03	
	0.02		0.02		0.02		0.04		18.41		17.52		14.91	
Water at handwashing place	0.03		− 0.04	*	− 0.06	***	0.06		50.85	**	− 25.69		− 27.38	
	0.02		0.02		0.02		0.04		19.17		18.24		15.54	
*VIF*	3.33		3.33		3.33		3.34		3.33		3.33		3.33	
*N*	1277		1277		1277		1277		1277		1277		1277	

The table lists first coefficients and below standard errors for each key independent variable. ***p < 0.001, **p < 0.01, *p < 0.05. Results for control variables are included in Table A2in the online Appendix

To expand on the last point, in contradiction of our first hypothesis, the pandemic—or more precisely, its first wave observed here—does not appear to have unequivocally worsened malnutrition rates amongst young Indian children. In the individual-level results (Table 2), the pandemic is linked with lower height-for-age scores but also with lower likelihood of children being wasted or anaemic, measures of more acute nutrition deprivation. In the district-level results (Table 3), the pandemic is linked with lower prevalence of stunting, underweight, wasting, and anaemia. However, it is not significantly associated with overall greater height-for-age, weight-for-age or weight-for-height, indicating that the effect may have been one of malnutrition amelioration rather than of an overall improvement in Indian children’s growth.

Looking at the role of the ICDS, which we hypothesised could have helped cushion the negative economic impact of the Covid-19 lockdown alongside other government welfare programmes, it is not positively connected with better nutrition outcomes. The results are largely insignificant with the exception of wasting and weight-for-height in the individual data, where receiving weekly food from the ICDS is associated with worse nutrition outcomes. The nature of the relationship in that case, however, is more likely in the opposite direction, with wasted children more likely to be recipients of the ICDS food aid than others. We investigate the relationship between the ICDS, children’s nutrition outcomes, and the pandemic in more depth further in the article but before doing so, let us discuss the other key independent variables.

From the dietary variables, consuming animal-sourced food—unlike the minimum acceptable diet—is evidently and consistently associated with lower malnutrition prevalence as well as with greater height and weight. This is in line with our second hypothesis. From the agricultural variables examined, having access to agricultural land is linked with lower likelihood of stunting and underweight. In contrast, cattle and poultry ownership are associated with worse nutrition outcomes. This might seem counterintuitive since households with cattle or poultry have more direct access to animal-sourced food. Nevertheless, it could be a function of a correlation between agricultural land and livestock ownership as well as of the higher exposure to animal-vector gastro-intestinal diseases in livestock-owning households^9^ [e.g., 11, 13].

WASH is also clearly linked with better nutrition outcomes, consistent with our third hypothesis. Households with private improved toilets are less likely to have stunted, underweight, wasted, and anaemic children and more likely to have children with greater height-for-age, weight-for-age, and weight-for-height scores. The same is true of the prevalence of private improved toilets on the district level—districts with better sanitation have better nutrition outcomes on most of the nutrition dimensions analysed. Access to an improved water source and water at handwashing place are also associated with some better nutrition outcomes—lower likelihood of wasting in the case of an improved water source and reduced prevalence of underweight and wasting in the case of water accessibility at handwashing place.

Before delving in more detail into the relationship between the pandemic and the other key variables, Tables A1 and A2 in the Online Appendix show the relationships between control variables and nutrition outcomes. These are largely in line with expectations. At the child level, boys are more likely to be malnourished than girls, as are higher-order children, children with shorter birth intervals with preceding siblings, children who were born prematurely, and children who have had diarrhoea or fever within two weeks of the survey. Wealthier households and more educated mothers are more likely to have well-nourished children; the opposite is true of children born to underweight and anaemic mothers. Upper-caste children are significantly less malnourished than children from other castes. Further area characteristics that lower malnutrition likelihood are rural and coastal areas of residence. The results from the individual-level data are largely replicated in the district-level data displayed in Table 3.

### The pandemic puzzle

**Fig. 2:**
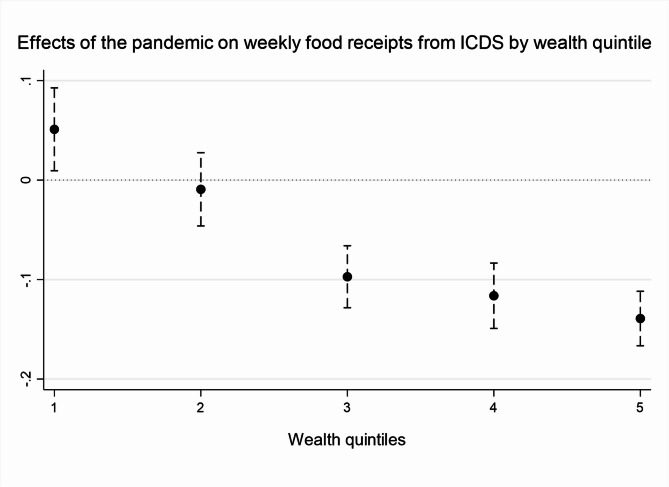
The pandemic’s effect on ICDS food receipts by household wealth quintile. *Source*: authors’ own analysis. Figure depicts the marginal effects of regressing the pandemic variable on ICDS weekly food receipts by household wealth quintile.

Our finding that the pandemic was not significantly associated with children’s malnutrition contrasts both with our original expectations and much of existing literature. The fact that the pandemic sometimes appears actually correlated with a reduced risk of malnutrition, particularly of wasting and anaemia, but not with increased children’s height or weight, raises the possibility that this effect could be driven by the expansion of welfare safety nets during the pandemic. The NFHS data contain information only on one relevant welfare programme, the ICDS. Table 4 below looks at the proportion of Indian households using the programme’s different services and shows that the weekly provision of food by the service declined after the first wave of Covid-19, although not drastically—from 51 percent in 2019 to 46 percent in 2021. Figure 2 further shows that after the first wave of Covid-19, food receipts from the programme became more progressively targeted—with the poorest 20 percent of households seeing a small but statistically significant increase in food benefits whilst the wealthiest 60 percent a significant reduction. Compared to food receipts, the provision of support during pregnancy and breastfeeding from the ICDS increased, from 88 and 80 percent before the pandemic to 91 percent and 88 percent after the first wave. This could be related to the increased use of mobile technology by ICDS workers following the onset of the pandemic, as described in previous research [51].

**Table 4:** ICDS provision in NFHS4 and 5, pre- and during the pandemic. *Source*: authors’ own analysis of NFHS data

	NFHS4		NFHS5 pre-pandemic		NFHS5 pandemic	
	*N*	%	*N*	%	*N*	%
Weekly food	39,315	50	23,946	51	12,060	46
Pregnancy support	39,315	81	23,946	85	12,060	91
Breastfeeding support	39,315	74	23,946	80	12,060	88

**Fig. 3:**
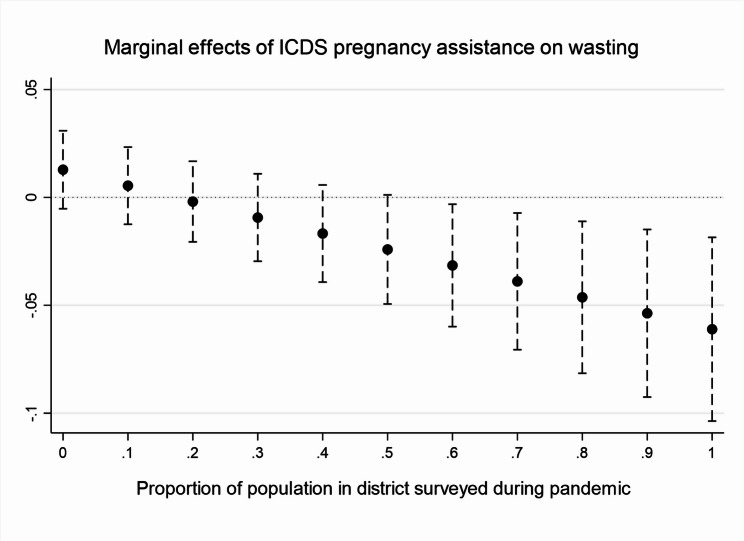
ICDS pregnancy. *Source*: authors’ own analysis. Figures depict the marginal effects of regressing ICDS assistance on wasting by the proportion of district surveyed during the pandemic (November 2020 to May 2021).

**Fig. 4:**
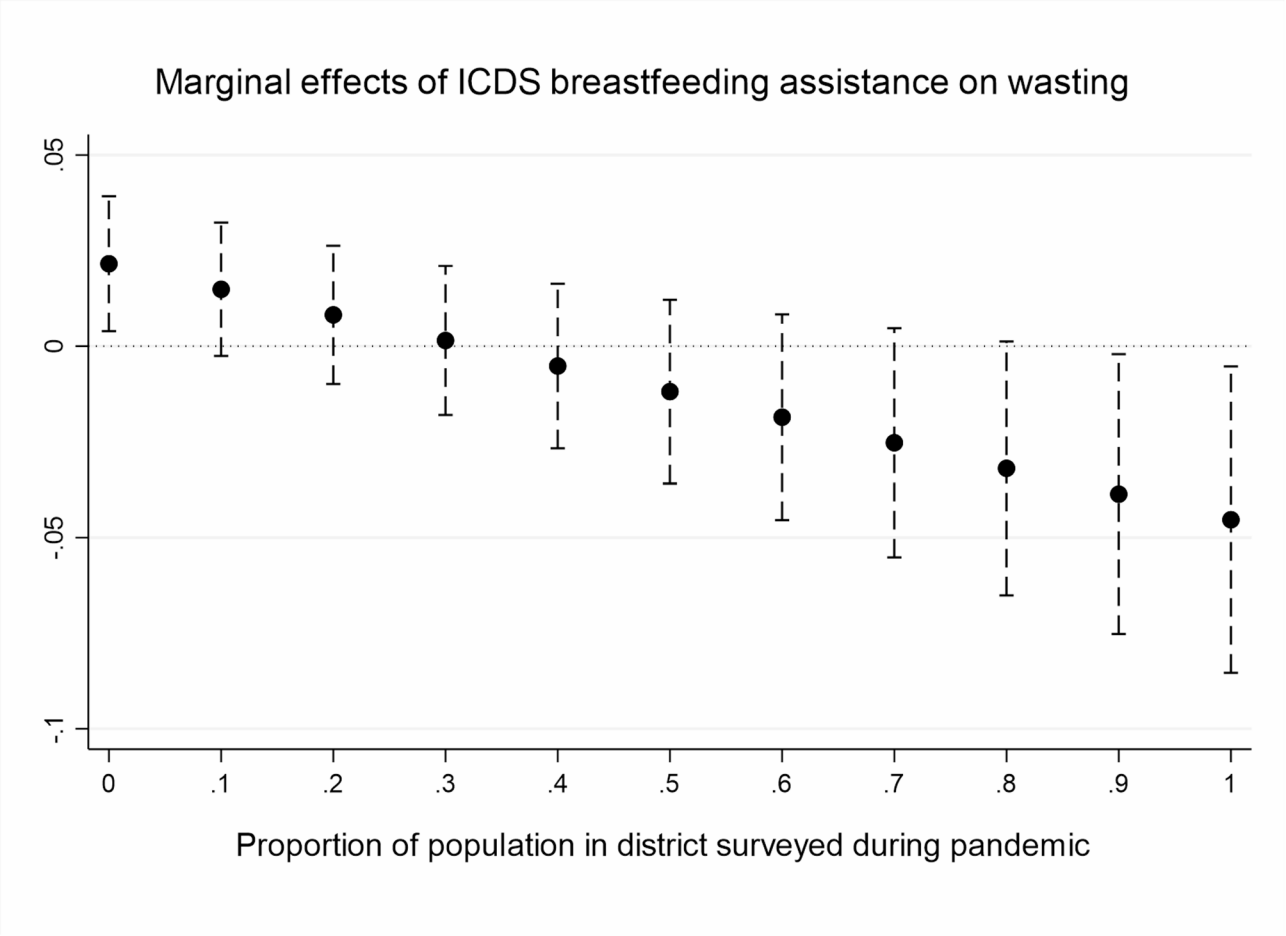
Breastfeeding support and wasting. *Source*: authors’ own analysis. Figures depict the marginal effects of regressing ICDS assistance on wasting by the proportion of district surveyed during the pandemic (November 2020 to May 2021).

Results in Table 5 and Figs. 3 and 4, which look at the links between ICDS pregnancy and breastfeeding support services, the pandemic, and children’s nutrition outcomes, interestingly demonstrate that after the first Covid-19 wave, the provision of regular pregnancy and breastfeeding support to mothers became linked with significantly lower rates of underweight, wasting, and anaemia when analysed through Probit regressions with interactions. This could potentially suggest that the increased provision of these ICDS services helped parents improve their children’s nutrition outcomes, through better feeding or sanitation practices.

**Table 5:** Links between ICDS services, the pandemic, and nutrition outcomes

	Assistance in pregnancy								Assistance while breastfeeding							
	*Stunted*		*Underweight*		*Wasted*		*Anaemic*		*Stunted*		*Underweight*		*Wasted*		*Anaemic*	
NFHS V	0.03	***	0.028	***	0.01		0.13	***	0.03	***	0.03	***	0.00		0.13	***
	0.01		0.010		0.01		0.04		0.01		0.01		0.01		0.04	
Pandemic	− 0.02		0.031		0.02		0.02		− 0.02		0.02		0.01		0.02	
	0.06		0.050		0.06		0.09		0.06		0.05		0.06		0.09	
ICDS assistance	0.03	*	0.052	***	0.02		− 0.03		0.03	*	0.06	***	0.02	*	− 0.02	
	0.02		0.020		0.02		0.08		0.02		0.02		0.01		0.06	
Pandemic* ICDS assistance	− 0.01		− 0.077	**	− 0.08	**	− 0.23	*	0.00		− 0.07	**	− 0.07	**	− 0.24	*
	0.04		0.030		0.04		0.09		0.15		2.82		2.87		0.09	

Partial results of regressions akin to those in Table 3 and A2, with a substitution of the ICDS variables (instead of ‘weekly from the ICDS’ ‘ICDS assistance in pregnancy’ and ‘ICDS assistance with breastfeeding’ are used) and addition of their interaction with the pandemic variable. The table lists first coefficients and below standard errors for each key independent variable. ***p < 0.001, **p < .01, *p < 0.05

However, as we explained earlier, the direction of the relationship between ICDS services and nutrition outcomes is endogenous—i.e., ICDS services are more likely to be claimed by less nutrition-secure households but might simultaneously help improve nutrition outcomes—and the effects of the programme are hence difficult to disentangle from its targeting. One common empirical approach that attempts to address this issue is Propensity Score Matching (PSM), a quasi-experimental method based on the construction of a synthetic control group to the ‘treated’ children based on observable characteristics [7]. The output indicators of the ‘constructed’ control group are subtracted from those of the treated group to try to ascertain the programme’s impact. Tables A3 and A4 in the online Appendix present results of PSM for ICDS weekly food receipts as treatment (Table A3), separately for NFHS4, NFHS5 pre-pandemic, and NFHS5 pandemic, and for ICDS pregnancy and breastfeeding assistance (Table A4), separately for NFHS5 pre-pandemic and pandemic. The results are largely insignificant, in all time periods, which aligns with some previously published findings [7, 67]. Nevertheless, even the PSM cannot perfectly address the endogenous relationship, as it controls only for households’ and individuals’ observable characteristics; yet unobservable characteristics also influence whether households access ICDS assistance. Qualitative data on the ICDS discussed in the comparative case study can thus shed more light on this issue.

Other possible, and more likely, factors underlying our observation of several positive associations between the pandemic variable and children’s nutrition outcomes are shown in Table 6. The table displays the prevalence of children’s diseases and improved-WASH variables in the districts that were not surveyed during the pandemic, in 2015/16 and 2019/20, as compared to the districts that were surveyed after the onset of the pandemic, again in 2015/16 and then in 2020/21. The occurrence of disease—both diarrhoea and fever within two weeks of the survey—appeared negatively correlated with children’s nutrition outcomes, particularly their weight, in our earlier results (Tables A1 and A2). Table 6 shows that following the first wave of Covid-19, Indian children were suffering significantly less from diarrhoea and fever, likely due to reduced social contact [55]. This is a very plausible pathway through which the pandemic may have contributed to reduced malnutrition rates. Another, related, pathway might be the greater importance placed on good-quality WASH in the pandemic messaging. Table 6 shows that whilst there was a general increase in the presence of water and soap/ash at handwashing places between NFHS 4 and 5, the increase was significantly greater in districts surveyed after the first wave of Covid-19. Since these variables are also linked with lower malnutrition rates (Tables A1 and A2), this is another way through which the Covid-19 pandemic might have translated in some cases into lower children’s malnutrition. The following comparative study of Rajasthan and Himachal Pradesh explores these issues in greater depth.

**Table 6:** Disease and WASH changes from NFHS 4–5 for districts by pandemic status. *Source*: authors’ own analysis of NFHS data

	NFHS 4, non-pandemic districts (%)	NFHS 5, non-pandemic districts (%)	NFHS 4, pandemic districts (%)	NFHS 5, pandemic districts (%)
Fever	11.85	15.67	14.34	7.63
Diarrhoea	7.59	7.90	10.03	5.33
Water at handwashing place	86.90	90.77	83.94	91.20
Soap or ash at handwashing place	73.59	77.26	73.18	80.79
Private improved toilet	44.35	65.26	36.38	64.76

### Comparative case study—Rajasthan and Himachal Pradesh

**Fig. 5:**
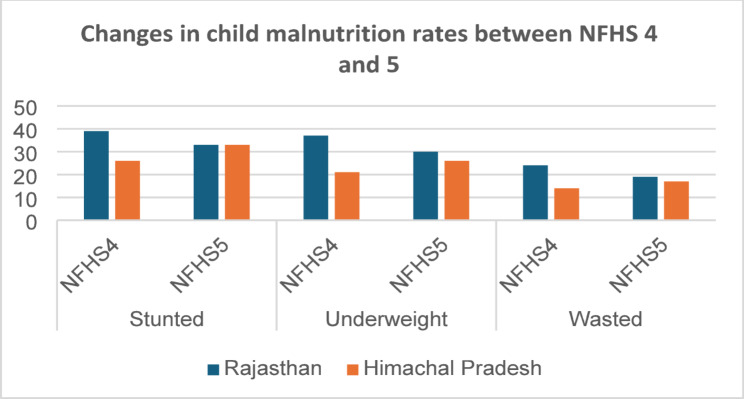
Malnutrition rates in Rajasthan and Himachal Pradesh. *Source*: authors’ own analysis of NFHS data

To gain a deeper understanding of the factors underlying the trajectory of change in Indian children’s nutrition outcomes, this second part of our analysis compares the recent experience of two states—Rajasthan and Himachal Pradesh. We chose these two states because, as Fig. 5 shows, whilst Rajasthan experienced a significant reduction in child malnutrition rates between NFHS4 and 5, the opposite happened in Himachal. The proportion of under-five stunted, underweight, and wasted children in Rajasthan declined from 39, 37, and 24 percent in 2015/16 to 33, 30, and 19 percent in 2019/21; conversely, in Himachal the corresponding rates increased from 26, 21, and 14 percent to 33, 26, and 17 percent. The overall malnutrition rates in Rajasthan have remained slightly higher than in Himachal but the two state’s trajectories have been very different, making them good case studies for a more detailed study of the relevant factors.

Before delving into that analysis, we present some basic characteristics of the two states in Table 7. In some respects, the states are quite different. Himachal Pradesh is only one sixth of Rajasthan in size, has one tenth of Rajasthan’s population, and has a significantly larger proportion of migrant labourers (35.2 percent compared to 7.6 percent of the population) [68]. However, there are also many similarities between the states. They are both situated in northern India, not far from the country’s capital, New Delhi. They are both predominantly Hindu, with relatively small Muslim minorities, and most people in both states speak Hindi. They are also mostly rural, with only 7 percent of Himachal and 17 percent of Rajasthan inhabitants residing in urban areas.

**Table 7:** Rajasthan and Himachal—Selected characteristics. *Source*: 2, 3, 68

	Himachal Pradesh	Rajasthan
Land size (km2)	55,673	342,269
Population (mill)	6.9	68.5
GDP per capita (USD)	2774	1787
Muslim population (prop.)	2.5%	9.0%
Urban population (prop.)	7.0%	17.3%
Migrant population (prop.)	35.2%	7.6%

#### Welfare programmes

In turn, we now consider in more detail the factors identified in the previous section as influential for children’s nutrition outcomes—the pandemic, social welfare programmes, dietary factors, WASH, and their interaction. An important point to note here is that NFHS 5 data for Himachal Pradesh were all gathered prior to the onset of the pandemic, whereas 15 out of 33 districts in Rajasthan were partly or wholly surveyed after the first wave of Covid-19. Hence, whilst the NFHS5 data for Rajasthan partially reflect the effects of the pandemic, the data for Himachal Pradesh do not.

The first factor that we investigated in the quantitative section as a pathway through which the pandemic may have influenced malnutrition rates were welfare programmes, specifically the ICDS, on which the NFHS gathers data. Our data analysis did not show a clear link between the programme’s assistance and better outcomes, but some researchers found the ICDS to have a positive impact on nutrition outcomes, at least for some groups of children [37–39]. In both Rajasthan and Himachal, the ICDS coverage increased between NFHS4 and NFHS5, particularly when it comes to pregnancy and breastfeeding assistance—from 78 and 65 percent to 84 and 80 percent in Himachal and from 49 and 38 percent to 68 and 57 percent in Rajasthan. But in Rajasthan, the increase in the coverage by these services was particularly notable during the pandemic, with a 39-percent increase for the coverage of pregnancy assistance and 61-percent for the coverage of breastfeeding assistance (the proportion of households receiving weekly food rations declined, on the other hand, from 55 to 47 percent, but as on the national level, this affected households in the poorest quintile proportionally the least). A similar enlargement of ICDS pregnancy and breastfeeding services occurred according to our interviewees during the pandemic in Himachal—had survey data there been collected also after the first wave of Covid-19, perhaps the results for malnutrition rates in Himachal would have looked different.

Further innovations were recently made in the Rajasthan ICDS, which might have also helped with the state’s significant stride forward in nutrition outcomes. One has been the rollout of the community-based management of malnutrition in 20 Rajasthani districts in 2018 [69]. The programme surveyed all children between 6 and 59 months old in the districts where it was implemented and referred children affected by severe acute malnutrition to medical centres or community care, with good results. Sixty-seven percent of the treated children recovered within 12 weeks—without the programme, they would have likely continued to be malnourished. The other improvement within the Rajasthani ICDS has been the establishment of specific coordination between the Ministry of Women and Children, traditionally tasked with overseeing the ICDS, and the Ministry of Education, to ensure that the activities of both ministries aligned in their support for the ICDS. One result of this coordination has been greater awareness of early child development issues throughout the state’s government; another result has been the increasing trend of opening Anganwadi centres in primary schools, a move that boosted Anganwadi attendance by younger siblings of primary school pupils. We are not aware of equivalent initiatives in Himachali ICDS.

The other national programme with a large potential to affect children’s nutrition is the PDS. Its coverage in Rajasthan, estimated at 61 percent, is greater than in Himachal (38 percent) [70] and according to our interviewees, Rajasthan has made good progress in reducing the programme’s leakage by eliminating intermediary godowns (storage) for grains. Now the PDS grains are transferred directly from national godowns to fair-price shops in Rajasthan, with digital tracking of the deliveries and immediate online reporting of all transactions, until the delivery to the household [71]. The PDS offering in Himachal Pradesh is much more comprehensive than in Rajasthan, on the other hand. In addition to wheat and rice, Himachali government provides NFSA beneficiaries also with subsidised (not free) edible and refined oil, salt, sugar, and pulses [72] and the food is available at subsidised, albeit less so, prices also to Himachali residents without NFSA ration cards [72]. According to interviewees, the issue of the quality of food available through the PDS is still problematic in both states, which more localised quality controls could help address [71, 72].

However, the biggest apparent problem with the PDS in Himachal has been the issue of migrant labourers. As Table 7 shows, 35 percent of Himachali population is constituted by labour migrants. Whilst these are predominantly men, some are accompanied by women and children—and unless they have family in Himachal Pradesh, they are not entitled to obtain a Himachali ration card (either an NFSA or a general one). The Indian government has tried to address this problem recently with the One Nation One Ration Card programme, but thus far this has been largely not functional in Himachal and hence migrant labourers have not been able to obtain their PDS rations whilst living in Himachal [70]. The operation of the One Nation One Ration Card Scheme in Rajasthan was in contrast described as relatively successful [71]—but even if it were not, Rajasthan is home to a significantly smaller proportion of migrant labour than Himachal, which makes the issue of a lack of access to the PDS due to changed area of residence less problematic on the state level.

In response to the Covid-19 pandemic, the Indian government temporarily expanded the PDS with the PMGKAY, which provided NFSA beneficiaries with 5 kg extra grains per person and 1 kg of pulses per household per month between March 2020 and December 2023. Unlike the ICDS, the operation of which was disrupted due to the closure of Anganwadi centres during the first lockdown, the PDS along with the PMGKAY operated relatively smoothly throughout most of the pandemic. The granular data on Rajasthani PDS deliveries demonstrate that there were some leakages of the rations, but most households received most of their grain and pulse entitlements.

#### Dietary trends, WASH, and disease

However, even though the safety-net programmes, PDS and ICDS, were relatively resilient throughout the pandemic, it has long been agreed that their positive influence on children’s nutrition outcomes is limited by the low diversity of the food they provide. They are particularly low on protein-rich and animal-sourced food, which the quantitative analysis confirmed as one of the key drivers of Indian children’s nutrition outcomes. The consumption of animal-sourced food by Indian children is in general very low by international standards. It increased between NFHS4 and 5 but only slightly, from 35 percent of 6–23 month-old-children having eaten any ASF in the last 24 h before the survey in 2015/16 to 39 percent in 2019/21. The rate of increase was dampened by the pandemic, which was undoubtedly also connected with the low or non-existent provision of animal-sourced food by the PDS and ICDS, for cultural and religious reasons. In recognition of the situation, in recent years some Indian states have started to include eggs in ICDS meals, but neither HP nor Rajasthan do so [71–73]. HP has trialled including eggs but due to some parental protest terminated the pilot [72]. Rajasthan has not even attempted to include eggs but did start to include greater amounts of dairy, which unlike meat or eggs does not face religious objections. Himachali ICDS meals also include a small amount of milk powder, but the protein content of that amount is negligible and other options, such as providing paneer, have been rejected on hygiene grounds, as most Anganwadi centres lack refrigerated storage facilities [72].

Looking to WASH, another key driver of child nutrition outcomes, both Rajasthan and Himachal Pradesh experienced improvements in WASH access between NFHS4 and NFHS5. Particularly the access to private improved toilets grew significantly and more so in Rajasthan than in Himachal. Whilst the proportion of households with a private improved toilet in Himachal rose from 68 to 78 percent (a 15-percent increase), in Rajasthan the proportion expanded from 38 to 64 percent (a 68-percent increase) [2, 3]. The other WASH and disease variables highlighted as potential pathways through which the pandemic may have reduced some malnutrition rates changed in the desirable direction during the pandemic also in Rajasthan—the rates of households with water and soap/ash at handwashing points increased and the rate of diarrhoea and fever in young children decreased. Our interviewees in both Himachal and Rajasthan also noted the lower disease occurrence in young children during the pandemic, which may have contributed to better nutrition outcomes even during a time of relative economic crisis [70, 71].

## Discussion and conclusions

Child nutrition outcomes specifically and food and nutrition security more broadly remain a challenge in India. Whilst there have been improvements in recent decades, the rate of progress has been slow. Our study has contributed to the understanding of the multiple drivers of these complex issues and underscored the importance of child feeding practices, including animal-sourced food consumption, of improved water, sanitation, and hygiene practices, and of the resilience of relevant welfare programmes, which had been tested during the pandemic. The study also highlighted that the effects of the pandemic on India’s nutrition security have not been uniformly as negative as suggested in other existing research and, through the comparative qualitative case study of Rajasthan and Himachal Pradesh, underscored the importance of locally tailored state-level policies. More broadly, the study contributes to global literature on the impacts of COVID-19 by offering evidence that, under certain conditions, social protection systems and improved public health behaviours can buffer vulnerable populations from expected nutritional declines during crises.

The importance of timely weaning of children with sufficiently diverse and frequent diets for Indian children’s nutrition outcomes has been previously established [7]. This study further emphasises the importance of introducing proteins and micronutrients found in ASFs into children’s diets to encourage growth and reduce malnutrition rates. Our interviews with civil servants in Rajasthan and Himachal revealed that there is awareness of this issue on the ground but there are barriers still in place to increasing access to such food in welfare programmes, cultural, religious, as well as logistic, including a lack of refrigerated storage spaces. Greater emphasis on parental awareness-raising in this regard could make a big difference, too, as even many non-vegetarian parents do not feed ASFs to young children due to erroneous beliefs about potential harm [74]. Neither Rajasthan nor Himachal Pradesh have thus far included eggs in their ICDS offering, unlike many North-Eastern and Southern Indian states, but our interviewees in Himachal suggested that since the Covid-19 pandemic, there has been a greater general acceptance of egg consumption even by vegetarians [71]. The state and local governments could thus make a stronger case for the inclusion of ASFs in feeding programmes, which could contribute to healthier children. Unlike Himachal, Rajasthan has included more dairy in its ICDS food, which could be one of the reasons why Rajasthan has managed to reduce child malnutrition rates quite notably between 2015–16 and 2019–21.

Our study further re-affirmed the importance of access to improved WASH in attaining positive nutrition outcomes. At all levels of the data analysis, ownership of private improved toilets was significantly positively associated with lower levels of child malnutrition. This was true not only at the individual level. Districts with greater prevalence of private improved toilets were found to have further positive association with lower levels of malnutrition, highlighting the importance of communal or ecological sanitation and hygiene as well. The Swachh Bharat programme undoubtedly increased both people’s access to and usage of improved sanitation; however, at the communal level the question of sewage disposal needs further addressing [42]. Both Rajasthan and Himachal Pradesh experienced an increase in people’s access to improved sanitation as well, but the rate of increase in Rajasthan has been much steeper, which could be another reason underlying the divergence in the two states’ child-nutrition trajectories.

Finally, the study shed some new light on the effects of the pandemic on food and nutrition security in India and its interaction with the welfare services in place. Unlike other existing studies, the quantitative data analysis, which took advantage of the fact that part of the NFHS 5 data were gathered after the first wave of the Covid-19 pandemic, did not reveal a consistently negative picture of the pandemic’s effects. The results did not suggest that the pandemic had an overall positive effect on children’s growth but showed several positive associations between the pandemic and some malnutrition measures, particularly wasting and underweight. One potential explanation for these results might lie in India’s welfare programmes in place and their expansion during the pandemic. The PDS was during the pandemic supplemented with the PMGKAY, which gave NFSA-eligible households extra grains and pulses. The ICDS operations were disrupted more but home food deliveries were introduced after initial disruption and the provision of some services, such as the ICDS pregnancy and breastfeeding advice, increased, also because of greater utilisation of mobile technologies [see also 75]. However, as our quantitative and qualitative analysis indicated, there are other, more likely pathways through which the pandemic’s negative nutrition effects may have been successfully mitigated. Hygiene observation (e.g., handwashing with soap) during the pandemic improved, which, together with lower levels of social contact, led to a lower prevalence of diseases (diarrhoea, fever) in young children, which are linked with lower malnutrition rates.^10^

That is not to contend, however, that the performance of welfare programmes and their local tailoring have had no bearing on the trajectory of child malnutrition rates across India, as was underscored by our comparative case study of Rajasthan and Himachal. Some of the reasons why the nutrition situation in Rajasthan improved between 2015–16 and 2019–21 and worsened in Himachal in the same time period were undoubtedly linked with the performance of the PDS and ICDS. In Rajasthan, the improvements may have been at least partially driven by better integration and coordination of the Education and Women and Child ministries in the delivery of ICDS services as well as innovative programmes like the community-based management of malnutrition. Conversely, in Himachal Pradesh the deterioration was likely related to a rapid influx of migrant labourers, which have had a hard time gaining access to PDS rations if migrating from other states. The One Nation One Ration Card initiative is aimed at addressing this specific issue but so far, its implementation has been patchy and largely inadequate, particularly given India’s growing rate of inter-state labour movement [70, 71].

Our findings should be interpreted in light of several limitations. Due to the largely cross-sectional nature of the NFHS data, our quantitative results do not lend themselves to direct causal inference. The NFHS further lacks information on welfare programmes beyond the ICDS, limiting our ability to evaluate the impact of other relevant interventions—such as the PDS—within the quantitative analysis. As a result, insights into these programmes are drawn primarily from our qualitative data, which, due to time and resource constraints, were collected in only two Indian states. In addition, our quantitative analysis of the pandemic’s influence is confined to the period covered by NFHS5, which concluded in May 2021, before the onset of India’s more severe second wave of Covid-19. The nutritional consequences of that later phase remain to be assessed, pending the release of NFHS6 data.

In conclusion, India’s slow progress in improving child nutrition outcomes despite rapid economic growth underscores the complexity of malnutrition and the need for multifaceted solutions. Our study’s findings highlight the critical role of ASF consumption, improved WASH services, and resilient welfare programmes. The comparative analysis of Rajasthan and Himachal Pradesh provided further insight into issues of effective policy implementation and highlighted the importance of state-specific strategies. Concerted efforts at both Indian national and state levels, informed by robust data^11^ and innovative practices, are essential to accelerating progress in improving India’s child nutrition outcomes.

## Supplementary Information

Below is the link to the electronic supplementary material.

Supplementary Material 1

## Data Availability

Datasets analysed are publicly available at https://microdata.worldbank.org/index.php/catalog/3110 and at https://microdata.worldbank.org/index.php/catalog/4482.

## Abbreviations

ASF: Animal–sourced food
DHS: Demographic and health surveys
HP: Himachal Pradesh
ICDS: Integrated child development services
LMICs: Low–and middle–income countries
MAD: Minimum acceptable diet
MDMS: Mid–day meal scheme
NFHS: National Family Health Survey
NFSA: National Food Security Act
PDS: Public distribution system
PSM: Propensity score matching
PMGKAY: Pradhan Mantri Garib Kalyan Anna Yojana
USAID: United States Agency for International Development
VIF: Variance inflation factor
WASH: Water, sanitation, and hygiene
WHO: World Health Organisation

## Footnotes

1 Since 2020, the subsidy for the 5 kg per month is 100%; i.e., the 5 kg are free.

2 This programme is of less interest here as our main focus are nutrition outcomes amongst pre-school-aged children.

3 A panel could not be constructed since each round of NFHS surveys different households. As a robustness test, data are also analysed separately for each wave.

4 All statistical estimations were conducted using the statistical software STATA 18 MP.

5 Our reasons for the case selection are explained in greater detail in the case-study section of the article.

6 The term ‘nutrition outcome’ used in this article covers children’s outcomes on height, weight, and blood red cells; ‘malnutrition’ relates to nutrition outcomes in these areas that are below two standard deviations (< 1.96 Z score) from the WHO median—these include stunting, underweight, wasting, and anaemia.

7 Southern states (Andaman and Nicobar Islands, Andhra Pradesh, Karnataka, Kerala, Lakshadweep, Puducherry, Tamil Nadu, Telangana), North-Eastern states (Arunachal Pradesh, Assam, Manipur, Meghalaya, Mizoram, Nagaland, Sikkim, Tripura), Eastern states (Bihar, Jharkhand, Odisha, West Bengal), Northern states (Chandigarh, Haryana, Himachal Pradesh, Jammu and Kashmir, Ladakh, Delhi, Punjab, Rajasthan), Central states (Chhattisgarh, Madhya Pradesh, Uttar Pradesh, Uttarakhand), and Western states (Dardra, Nagar Haveli, Daman, and Diu, Goa, Gujarat, Maharashtra).

8 The Variance Inflation Factors (VIF) in all models in Tables 2 and 3 are below 5, assuaging concerns about potential multicollinearity.

9 Indeed, if we re-estimate regressions from Table 2 models 2, 4, 6, and 8 without the ‘agricultural land’ variable, most of the significant positive associations between livestock ownership and child malnutrition measures disappear—except for the association between wasting and poultry ownership, highlighting the possibility that particularly poultry ownership might be linked with higher incidence of gastro-intestinal diseases. This aligns with previous findings [e.g., 7, 13].

10 However, it is likely that these improvements were at least partly temporary, as both disease incidence and poor hygiene practices may have resurged once the immediate pressures of the pandemic subsided.

11 At the time of writing, the US has cancelled funding for all Demographic and Health Surveys, on which the NFHS is also based. The authors hope that India, which is now self-funding the 6th round of NFHS, will follow previous practice of releasing the final microdata to academic researchers for analysis. The cancellation of the DHS is to grave detriment to global health research, particularly in low- and middle-income countries, and should be resurrected through other financial means.
