# Supplementary material for: Progress in child nutrition outcomes: insights from India’s recent experience
Source: BMC Public Health. 2025 Sep 30;25:3179. doi: 10.1186/s12889-025-24436-y (PMC12487531; doi:10.1186/s12889-025-24436-y)
Supplement: Supplementary file 1 — Supplementary Material 1 [file 12889_2025_24436_MOESM1_ESM.docx]

**Appendix**

**Table A1.** **NFHS4 and 5 individual-level results – control variables**

|  | **Stunted** | | | | **Underweight** | | | | **Wasted** | | | | **Anaemic** | | | | **ZHA** | | **ZWA** | | **ZWH** | |
| --- | --- | --- | --- | --- | --- | --- | --- | --- | --- | --- | --- | --- | --- | --- | --- | --- | --- | --- | --- | --- | --- | --- |
|  | (1) | | (2) | | (3) | | (4) | | (5) | | (6) | | (7) | | (8) | | (9) | | (10) | | (11) | |
| Male child | 0.26 | *** | 0.26 | *** | 0.26 | *** | 0.27 | *** | 0.12 | *** | 0.12 | *** | 0.09 | *** | 0.09 | *** | -22.92 | *** | -16.62 | *** | -6.67 | *** |
|  | 0.02 |  | 0.02 |  | 0.02 |  | 0.02 |  | 0.02 |  | 0.02 |  | 0.02 |  | 0.02 |  | 1.39 |  | 0.94 |  | 1.06 |  |
| Age (6-11 months - reference) |  |  |  |  |  |  |  |  |  |  |  |  |  |  |  |  |  |  |  |  |  |  |
| 12-17 months | 0.63 | *** | 0.63 | *** | 0.20 | *** | 0.19 | *** | -0.10 | *** | -0.11 | *** | 0.09 | *** | 0.09 | *** | -55.47 | *** | -12.91 | *** | -2.20 |  |
|  | 0.02 |  | 0.02 |  | 0.02 |  | 0.02 |  | 0.02 |  | 0.02 |  | 0.02 |  | 0.02 |  | 1.83 |  | 1.27 |  | 1.49 |  |
| 18-23 months | 1.03 | *** | 1.02 | *** | 0.45 | *** | 0.44 | *** | -0.27 | *** | -0.28 | *** | -0.03 |  | -0.04 |  | -93.64 | *** | -30.38 | *** | 1.24 |  |
|  | 0.02 |  | 0.02 |  | 0.02 |  | 0.02 |  | 0.02 |  | 0.02 |  | 0.02 |  | 0.02 |  | 1.95 |  | 1.27 |  | 1.55 |  |
| Preterm | 0.10 | ** | 0.10 | *** | 0.11 | *** | 0.10 | *** | 0.05 |  | 0.04 |  | 0.02 |  | 0.02 |  | -8.00 | ** | -5.71 | ** | -2.06 |  |
|  | 0.03 |  | 0.03 |  | 0.03 |  | 0.03 |  | 0.03 |  | 0.03 |  | 0.04 |  | 0.03 |  | 2.76 |  | 1.86 |  | 2.34 |  |
| Breastfed until 6 months | 0.04 |  | 0.05 |  | 0.12 | *** | 0.12 | *** | 0.09 | ** | 0.08 | ** | 0.16 | *** | 0.17 | *** | -8.32 | *** | -10.83 | *** | -9.87 | *** |
|  | 0.03 |  | 0.03 |  | 0.03 |  | 0.03 |  | 0.03 |  | 0.03 |  | 0.03 |  | 0.03 |  | 2.25 |  | 1.59 |  | 1.74 |  |
| Diarrhoea last 2 weeks | -0.01 |  |  |  | 0.10 | *** |  |  | 0.09 | ** |  |  | 0.01 |  |  |  | -3.22 |  | -4.92 | ** | -5.90 | ** |
|  | 0.02 |  |  |  | 0.02 |  |  |  | 0.03 |  |  |  | 0.03 |  |  |  | 2.11 |  | 1.52 |  | 1.80 |  |
| Fever last 2 weeks |  |  | -0.01 |  |  |  | 0.10 | *** |  |  | 0.10 | *** |  |  | -0.01 |  | -0.87 |  | -5.82 | *** | -8.04 | *** |
|  |  |  | 0.02 |  |  |  | 0.02 |  |  |  | 0.03 |  |  |  | 0.03 |  | 1.90 |  | 1.37 |  | 1.62 |  |
| Birth order | 0.07 | *** | 0.07 | *** | 0.08 | *** | 0.08 | *** | 0.03 | ** | 0.03 | * | 0.02 | * | 0.02 | * | -6.89 | *** | -6.58 | *** | -4.09 | *** |
|  | 0.01 |  | 0.01 |  | 0.01 |  | 0.01 |  | 0.01 |  | 0.01 |  | 0.01 |  | 0.01 |  | 0.78 |  | 0.55 |  | 0.63 |  |
| Birth interval with preceding sibling | 0.00 | *** | 0.00 | *** | 0.00 | *** | 0.00 | *** | 0.00 | ** | 0.00 | *** | 0.00 |  | 0.00 |  | 0.18 | *** | 0.13 | *** | 0.06 | * |
|  | 0.00 |  | 0.00 |  | 0.00 |  | 0.00 |  | 0.00 |  | 0.00 |  | 0.00 |  | 0.00 |  | 0.03 |  | 0.02 |  | 0.03 |  |
| Mother age at birth | -0.01 | *** | -0.01 | *** | -0.01 | * | -0.01 | * | 0.01 | * | 0.01 | ** | 0.00 |  | 0.00 |  | 1.01 | *** | 0.61 | *** | 0.06 |  |
|  | 0.00 |  | 0.00 |  | 0.00 |  | 0.00 |  | 0.00 |  | 0.00 |  | 0.00 |  | 0.00 |  | 0.22 |  | 0.15 |  | 0.18 |  |
| Education level (did not complete primary - reference) |  |  |  |  |  |  |  |  |  |  |  |  |  |  |  |  |  |  |  |  |  |  |
| Completed primary | -0.06 | * | -0.06 | * | -0.11 | *** | -0.11 | *** | -0.08 | ** | -0.08 | ** | -0.06 |  | -0.06 |  | 4.42 |  | 6.50 | *** | 4.22 | * |
|  | 0.02 |  | 0.02 |  | 0.03 |  | 0.03 |  | 0.03 |  | 0.03 |  | 0.03 |  | 0.04 |  | 2.25 |  | 1.60 |  | 2.03 |  |
| Completed secondary | -0.28 | *** | -0.28 | *** | -0.28 | *** | -0.27 | *** | -0.10 | *** | -0.09 | *** | -0.07 | * | -0.07 | * | 19.67 | *** | 16.89 | *** | 8.16 | *** |
|  | 0.02 |  | 0.02 |  | 0.02 |  | 0.02 |  | 0.03 |  | 0.03 |  | 0.03 |  | 0.03 |  | 1.99 |  | 1.50 |  | 1.87 |  |
| Tertiary | -0.46 | *** | -0.45 | *** | -0.55 | *** | -0.54 | *** | -0.23 | *** | -0.23 | *** | -0.10 | * | -0.12 | * | 35.01 | *** | 34.10 | *** | 19.55 | *** |
|  | 0.04 |  | 0.04 |  | 0.04 |  | 0.04 |  | 0.04 |  | 0.04 |  | 0.05 |  | 0.05 |  | 3.18 |  | 2.11 |  | 2.52 |  |
| Mother underweight | 0.22 | *** | 0.22 | *** | 0.50 | *** | 0.49 | *** | 0.35 | *** | 0.34 | *** | 0.14 | *** | 0.14 | *** | -23.49 | *** | -34.39 | *** | -31.46 | *** |
|  | 0.02 |  | 0.02 |  | 0.02 |  | 0.02 |  | 0.02 |  | 0.02 |  | 0.02 |  | 0.02 |  | 1.58 |  | 1.08 |  | 1.34 |  |
| Mother anaemic | 0.09 | *** | 0.09 | *** | 0.15 | *** | 0.15 | *** | 0.07 | *** | 0.07 | *** | 3.23 | *** | 3.22 | *** | -13.10 | *** | -10.79 | *** | -6.48 | *** |
|  | 0.02 |  | 0.02 |  | 0.02 |  | 0.02 |  | 0.02 |  | 0.02 |  | 0.05 |  | 0.05 |  | 1.48 |  | 1.00 |  | 1.15 |  |
| HH size | -0.01 | * | -0.01 |  | -0.01 | ** | -0.01 | * | -0.01 |  | -0.01 |  | 0.01 |  | 0.00 |  | 0.26 |  | 0.49 | * | 0.32 |  |
|  | 0.00 |  | 0.00 |  | 0.00 |  | 0.00 |  | 0.00 |  | 0.00 |  | 0.00 |  | 0.00 |  | 0.34 |  | 0.23 |  | 0.27 |  |
| Number of children under 5 | 0.08 | *** | 0.09 | *** | 0.07 | *** | 0.07 | *** | 0.00 |  | 0.00 |  | 0.01 |  | 0.01 |  | -7.16 | *** | -4.38 | *** | -0.71 |  |
|  | 0.01 |  | 0.01 |  | 0.01 |  | 0.01 |  | 0.01 |  | 0.01 |  | 0.01 |  | 0.01 |  | 1.02 |  | 0.73 |  | 0.85 |  |
| Female-headed HH | 0.03 |  | 0.02 |  | -0.01 |  | -0.02 |  | -0.05 | * | -0.05 |  | -0.05 |  | -0.05 |  | -3.66 |  | 1.87 |  | 3.51 | * |
|  | 0.03 |  | 0.02 |  | 0.02 |  | 0.02 |  | 0.03 |  | 0.03 |  | 0.03 |  | 0.03 |  | 2.16 |  | 1.40 |  | 1.63 |  |
| Wealth quintile (I - reference) |  |  |  |  |  |  |  |  |  |  |  |  |  |  |  |  |  |  |  |  |  |  |
| II | -0.15 | *** | -0.15 | *** | -0.12 | *** | -0.11 | *** | -0.05 |  | -0.04 |  | -0.02 |  | -0.02 |  | 7.44 | *** | 7.60 | *** | 2.24 |  |
|  | 0.02 |  | 0.02 |  | 0.02 |  | 0.03 |  | 0.03 |  | 0.03 |  | 0.03 |  | 0.03 |  | 2.25 |  | 1.60 |  | 1.90 |  |
| III | -0.20 | *** | -0.20 | *** | -0.24 | *** | -0.23 | *** | -0.13 | *** | -0.12 | *** | -0.01 |  | -0.02 |  | 15.28 | *** | 16.42 | *** | 7.46 | *** |
|  | 0.03 |  | 0.03 |  | 0.03 |  | 0.03 |  | 0.03 |  | 0.03 |  | 0.04 |  | 0.04 |  | 2.40 |  | 1.74 |  | 2.03 |  |
| IV | -0.31 | *** | -0.31 | *** | -0.34 | *** | -0.32 | *** | -0.18 | *** | -0.17 | *** | -0.02 |  | -0.02 |  | 22.03 | *** | 22.07 | *** | 11.31 | *** |
|  | 0.03 |  | 0.03 |  | 0.03 |  | 0.03 |  | 0.04 |  | 0.04 |  | 0.04 |  | 0.04 |  | 2.59 |  | 1.92 |  | 2.37 |  |
| V | -0.47 | *** | -0.47 | *** | -0.54 | *** | -0.53 | *** | -0.31 | *** | -0.29 | *** | -0.06 |  | -0.06 |  | 34.66 | *** | 34.68 | *** | 19.63 | *** |
|  | 0.04 |  | 0.04 |  | 0.04 |  | 0.04 |  | 0.04 |  | 0.04 |  | 0.05 |  | 0.05 |  | 3.01 |  | 2.16 |  | 2.63 |  |
| Caste (reference upper caste) |  |  |  |  |  |  |  |  |  |  |  |  |  |  |  |  |  |  |  |  |  |  |
| scheduled caste | 0.29 | *** | 0.28 | *** | 0.33 | *** | 0.31 | *** | 0.13 | *** | 0.13 | *** | 0.05 |  | 0.05 |  | -20.20 | *** | -19.92 | *** | -11.04 | *** |
|  | 0.03 |  | 0.03 |  | 0.03 |  | 0.03 |  | 0.03 |  | 0.03 |  | 0.03 |  | 0.03 |  | 2.66 |  | 1.93 |  | 2.18 |  |
| scheduled tribe | 0.21 | *** | 0.20 | *** | 0.36 | *** | 0.34 | *** | 0.28 | *** | 0.26 | *** | 0.13 | ** | 0.12 | *** | -13.29 | *** | -20.19 | *** | -13.36 | *** |
|  | 0.04 |  | 0.04 |  | 0.04 |  | 0.04 |  | 0.04 |  | 0.04 |  | 0.04 |  | 0.04 |  | 3.16 |  | 2.53 |  | 2.77 |  |
| other backward caste | 0.15 | *** | 0.16 | *** | 0.19 | *** | 0.19 | *** | 0.10 | ** | 0.11 | *** | 0.01 |  | 0.01 |  | -10.93 | *** | -13.77 | *** | -9.46 | *** |
|  | 0.03 |  | 0.03 |  | 0.03 |  | 0.03 |  | 0.03 |  | 0.03 |  | 0.03 |  | 0.03 |  | 2.29 |  | 1.72 |  | 2.03 |  |
| Religion (reference Hindu) |  |  |  |  |  |  |  |  |  |  |  |  |  |  |  |  |  |  |  |  |  |  |
| Muslim | 0.09 | ** | 0.08 | ** | 0.03 |  | 0.00 |  | 0.03 |  | 0.02 |  | -0.01 |  | -0.01 |  | -0.97 |  | -0.98 |  | 2.47 |  |
|  | 0.03 |  | 2.66 |  | 0.03 |  | 0.13 |  | 0.04 |  | 0.04 |  | 0.03 |  | 0.03 |  | 2.80 |  | 2.12 |  | 2.52 |  |
| Other religion | -0.04 |  | -0.05 |  | -0.11 | * | -0.11 | * | -0.08 |  | -0.09 |  | -0.15 | ** | -0.14 | ** | 3.02 |  | 8.16 | ** | 3.32 |  |
|  | 0.04 |  | -1.12 |  | 0.05 |  | -2.15 |  | 0.05 |  | 0.05 |  | 0.05 |  | 0.05 |  | 3.52 |  | 3.10 |  | 3.44 |  |
| Urban | -0.03 |  | -0.05 |  | 0.07 | ** | 0.03 |  | 0.08 | ** | 0.07 | * | 0.05 | * | 0.06 | * | 4.81 | * | -0.81 |  | -2.46 |  |
|  | 0.03 |  | -1.90 |  | 0.02 |  | 1.29 |  | 0.03 |  | 0.03 |  | 0.03 |  | 0.03 |  | 2.39 |  | 1.58 |  | 1.89 |  |
| Coastal | -0.22 | *** | -0.22 | *** | -0.21 | *** | -0.23 | *** | -0.10 |  | -0.11 | * | -0.16 | ** | -0.17 | ** | 12.62 | ** | 13.04 | ** | 6.84 |  |
|  | 0.06 |  | -3.81 |  | 0.06 |  | -3.96 |  | 0.05 |  | 0.05 |  | 0.06 |  | 0.06 |  | 4.89 |  | 4.15 |  | 4.13 |  |
| Region (Southern - reference) |  |  |  |  |  |  |  |  |  |  |  |  |  |  |  |  |  |  |  |  |  |  |
| Northeastern | -0.21 | *** | -0.21 | *** | -0.61 | *** | -0.64 | *** | -0.61 | *** | -0.63 | *** | -0.48 | *** | -0.49 | *** | 9.00 |  | 44.15 | *** | 53.10 | *** |
|  | 0.06 |  | -3.45 |  | 0.07 |  | -9.10 |  | 0.07 |  | 0.07 |  | 0.07 |  | 0.07 |  | 5.19 |  | 4.39 |  | 4.92 |  |
| Eastern | -0.11 | * | -0.11 | * | -0.04 |  | -0.05 |  | -0.02 |  | -0.03 |  | -0.11 | * | -0.11 | * | 4.99 |  | 4.68 |  | 4.42 |  |
|  | 0.05 |  | -2.16 |  | 0.05 |  | -0.92 |  | 0.05 |  | 0.05 |  | 0.06 |  | 0.06 |  | 4.41 |  | 3.28 |  | 3.87 |  |
| Northern | -0.16 | ** | -0.13 | ** | -0.21 | *** | -0.16 | ** | -0.22 | *** | -0.20 | *** | 0.01 |  | 0.02 |  | 1.88 |  | 13.47 | *** | 17.43 | *** |
|  | 0.05 |  | -2.67 |  | 0.05 |  | -3.13 |  | 0.05 |  | 0.05 |  | 0.06 |  | 0.06 |  | 4.56 |  | 3.16 |  | 3.82 |  |
| Central | -0.08 |  | -0.06 |  | -0.06 |  | -0.02 |  | -0.03 |  | -0.01 |  | -0.07 |  | -0.05 |  | -2.07 |  | 0.22 |  | 2.60 |  |
|  | 0.05 |  | -1.16 |  | 0.04 |  | -0.49 |  | 0.05 |  | 0.05 |  | 0.05 |  | 0.05 |  | 4.35 |  | 2.96 |  | 3.56 |  |
| Western | 0.15 | ** | 0.19 | *** | 0.25 | *** | 0.28 | *** | 0.24 | *** | 0.25 | *** | 0.18 | ** | 0.19 | ** | -7.64 |  | -16.99 | *** | -12.81 | ** |
|  | 0.06 |  | 3.33 |  | 0.05 |  | 5.63 |  | 0.05 |  | 0.05 |  | 0.06 |  | 0.06 |  | 5.10 |  | 3.23 |  | 3.93 |  |
| *VIF* | 1.70 | | 1.71 | | 1.70 | | 1.71 | | 1.70 | | 1.71 | | 1.70 | | 1.71 | | 1.72 | | 1.72 | | 1.72 | |
| *N* | *75321* | | *73118* | | *76427* | | *74196* | | *75028* | | *72849* | | *77695* | | *75404* | | *73118* | | *74196* | | *72849* | |

The table lists first coefficients and below standard errors for each key independent variable. ***p<0.001, **p<.01, *p<0.05. Results for main independent variables are shown in Table 2 in the Results section.

**Table A2.** **District-level results from merged NFHS 4 and 5 data - control variables**

|  | **Stunted** | | **Underweight** | | **Wasted** | | **Anaemic** | | **ZHA** | | **ZWA** | | **ZWH** | |
| --- | --- | --- | --- | --- | --- | --- | --- | --- | --- | --- | --- | --- | --- | --- |
|  | (1) | | (2) | | (3) | | (4) | | (5) | | (6) | | (7) | |
| Preterm | -0.03 |  | 0.01 |  | 0.02 |  | -0.05 |  | 35.12 |  | 14.63 |  | 9.86 |  |
|  | 0.02 |  | 0.02 |  | 0.02 |  | 0.04 |  | 22.71 |  | 21.61 |  | 18.38 |  |
| Diarrhoea last 2 weeks | -0.06 |  | 0.02 |  | -0.05 |  | 0.10 |  | -61.24 |  | -81.31 | * | -72.28 | * |
|  | 0.04 |  | 0.04 |  | 0.04 |  | 0.07 |  | 39.94 |  | 38.00 |  | 32.34 |  |
| Breastfed until 6 m | 0.02 |  | 0.10 | ** | 0.02 |  | -0.27 | *** | -194.37 | *** | -230.97 | *** | -185.50 | *** |
|  | 0.03 |  | 0.03 |  | 0.03 |  | 0.06 |  | 32.37 |  | 30.80 |  | 26.28 |  |
| Mother age at birth | 0.00 |  | 0.00 |  | 0.00 |  | -0.01 | ** | 1.20 |  | 2.85 |  | 2.77 | * |
|  | 0.00 |  | 0.00 |  | 0.00 |  | 0.00 |  | 1.74 |  | 1.66 |  | 1.41 |  |
| Education level | -0.05 | *** | -0.05 | *** | -0.02 | * | -0.13 | *** | 6.87 |  | 3.73 |  | 4.15 |  |
|  | 0.01 |  | 0.01 |  | 0.01 |  | 0.01 |  | 7.48 |  | 7.11 |  | 6.05 |  |
| Mother underweight | 0.12 | *** | 0.32 | *** | 0.10 | ** | 0.06 |  | -118.82 | *** | -144.76 | *** | -143.86 | *** |
|  | 0.03 |  | 0.03 |  | 0.03 |  | 0.06 |  | 29.45 |  | 28.02 |  | 23.87 |  |
| Mother anaemic | 0.06 | *** | 0.14 | *** | 0.09 | *** |  |  | -50.64 | ** | -71.17 | *** | -63.73 | *** |
|  | 0.02 |  | 0.02 |  | 0.02 |  |  |  | 16.70 |  | 15.89 |  | 13.53 |  |
| HH size | 0.00 |  | 0.01 |  | -0.01 |  | 0.01 |  | 0.24 |  | 0.35 |  | -0.91 |  |
|  | 0.00 |  | 0.00 |  | 0.00 |  | 0.01 |  | 3.51 |  | 3.34 |  | 2.84 |  |
| No of children under 5 | 0.09 | *** | 0.03 | * | -0.01 |  | -0.12 | *** | -26.70 | * | -10.91 |  | -8.79 |  |
|  | 0.01 |  | 0.01 |  | 0.01 |  | 0.02 |  | 13.06 |  | 12.43 |  | 10.58 |  |
| Female headed HH | 0.09 | ** | -0.05 |  | -0.10 | *** | -0.03 |  | -44.17 |  | -2.96 |  | -1.44 |  |
|  | 0.03 |  | 0.03 |  | 0.03 |  | 0.05 |  | 26.84 |  | 25.54 |  | 21.72 |  |
| Wealth quintile | -0.03 | *** | -0.02 | ** | 0.00 |  | 0.03 | *** | 2.83 |  | 1.70 |  | 4.01 |  |
|  | 0.01 |  | 0.01 |  | 0.01 |  | 0.01 |  | 5.27 |  | 5.02 |  | 4.27 |  |
| Scheduled caste and tribes | 0.01 |  | 0.01 |  | 0.02 |  | 0.12 | *** | 15.38 |  | 14.23 |  | 10.23 |  |
|  | 0.01 |  | 0.01 |  | 0.01 |  | 0.02 |  | 13.00 |  | 12.37 |  | 10.54 |  |
| Other backward castes | 0.01 |  | 0.04 | ** | 0.04 | ** | 0.11 | *** | -2.79 |  | -20.61 |  | -17.76 |  |
|  | 0.01 |  | 0.01 |  | 0.01 |  | 0.03 |  | 13.21 |  | 12.57 |  | 10.70 |  |
| Muslim | 0.00 |  | -0.01 |  | 0.00 |  | 0.03 |  | 17.29 |  | 15.71 |  | 15.96 |  |
|  | 0.01 |  | 0.01 |  | 0.01 |  | 0.02 |  | 10.45 |  | 9.95 |  | 8.48 |  |
| Urban | 0.04 | ** | 0.05 | *** | 0.05 | *** | 0.10 | *** | -0.45 |  | -8.49 |  | -12.98 |  |
|  | 0.01 |  | 0.01 |  | 0.01 |  | 0.03 |  | 13.01 |  | 12.38 |  | 10.56 |  |
| Coastal | -0.03 | *** | -0.04 | *** | -0.03 | *** | -0.03 | * | -0.10 |  | 5.55 |  | 6.69 |  |
|  | 0.01 |  | 0.01 |  | 0.01 |  | 0.01 |  | 6.27 |  | 5.96 |  | 5.08 |  |
| Region (Southern - reference) |  |  |  |  |  |  |  |  |  |  |  |  |  |  |
| Northeastern | -0.02 |  | -0.09 | *** | -0.06 | *** | -0.08 | *** | -31.38 | ** | -6.92 |  | 2.22 |  |
|  | 0.01 |  | 0.01 |  | 0.01 |  | 0.02 |  | 9.75 |  | 9.28 |  | 7.92 |  |
| Eastern | -0.02 | * | -0.02 | * | -0.01 |  | 0.01 |  | -12.23 |  | -9.47 |  | -3.31 |  |
|  | 0.01 |  | 0.01 |  | 0.01 |  | 0.02 |  | 8.39 |  | 7.98 |  | 6.80 |  |
| Northern | -0.04 | *** | -0.06 | *** | -0.05 | *** | 0.05 | ** | -10.13 |  | -2.62 |  | 0.32 |  |
|  | 0.01 |  | 0.01 |  | 0.01 |  | 0.02 |  | 9.80 |  | 9.33 |  | 7.95 |  |
| Central | -0.01 |  | -0.02 | * | -0.03 | ** | 0.01 |  | -23.04 | * | -16.16 |  | -11.29 |  |
|  | 0.01 |  | 0.01 |  | 0.01 |  | 0.02 |  | 9.33 |  | 8.87 |  | 7.56 |  |
| Western | 0.00 |  | 0.02 | * | 0.04 | *** | 0.05 | ** | 13.40 |  | 1.02 |  | 0.06 |  |
|  | 0.01 |  | 0.01 |  | 0.01 |  | 0.02 |  | 9.28 |  | 8.83 |  | 7.53 |  |
| *VIF* | *3.33* | | *3.33* | | *3.33* | | *3.34* | | *3.33* | | *3.33* | | *3.33* | |
| *N* | *1277* | | *1277* | | *1277* | | *1277* | | *1277* | | *1277* | | *1277* | |

The table lists first coefficients and below standard errors for each key independent variable. ***p<0.001, **p<.01, *p<0.05. Results for main independent variables are shown in Table 3 in the Results section.

**Table A3. Weekly food receipts from ICDS and children’s nutrition outcomes**

|  | *N untreated off support* | *N untreated on support* | **Stunted** | | **Underweight** | | **Wasted** | | **Anaemic** | | **ZHA** | | **ZWA** | | **ZWH** | |
| --- | --- | --- | --- | --- | --- | --- | --- | --- | --- | --- | --- | --- | --- | --- | --- | --- |
|  | *N treated off support* | *N treated on support* |  |  |  |  |  |  |  |  |  |  |  |  |  |  |
| **NFHS4** |  |  |  |  |  |  |  |  |  |  |  |  |  |  |  |  |
| *Nearest neighbour* | *0* | *18935* | 0.04 | *** | 0.01 |  | 0.01 |  | 0.00 |  | -9.98 | ** | -8.79 | ** | -5.16 |  |
|  | *10* | *19196* | 0.01 |  | 0.01 |  | 0.01 |  | 0.01 |  | 4.12 |  | 2.88 |  | 3.34 |  |
| *Five nearest neighbours* | *0* | *18935* | 0.03 | ** | 0.01 |  | 0.01 |  | 0.00 |  | -5.63 |  | -5.65 | ** | -3.97 |  |
|  | *10* | *19196* | 0.01 |  | 0.01 |  | 0.01 |  | 0.01 |  | 3.56 |  | 2.50 |  | 2.86 |  |
| **NFHS5 pre-pandemic** |  |  |  |  |  |  |  |  |  |  |  |  |  |  |  |  |
| *Nearest neighbour* | *0* | *10744* | 0.02 |  | -0.01 |  | -0.01 |  | -0.01 |  | -3.03 |  | -0.56 |  | 2.38 |  |
|  | *141* | *10616* | 0.02 |  | 0.01 |  | 0.01 |  | 0.01 |  | 6.39 |  | 4.09 |  | 5.12 |  |
| *Five nearest neighbours* | *0* | *10744* | 0.01 |  | 0.00 |  | -0.01 |  | 0.00 |  | -0.82 |  | -0.82 |  | 0.19 |  |
|  | *141* | *10616* | 0.01 |  | 0.01 |  | 0.01 |  | 0.01 |  | 5.31 |  | 3.43 |  | 4.28 |  |
| **NFHS5 pandemic** |  |  |  |  |  |  |  |  |  |  |  |  |  |  |  |  |
| *Nearest neighbour* | *0* | *5898* | -0.01 |  | 0.02 |  | 0.02 |  | -0.01 |  | -2.35 |  | -2.59 |  | -2.28 |  |
|  | *2* | *5038* | 0.02 |  | 0.02 |  | 0.01 |  | 0.02 |  | 6.51 |  | 4.53 |  | 5.42 |  |
| *Five nearest neighbours* | *0* | *5898* | 0.00 |  | 0.01 |  | 0.01 |  | 0.00 |  | -1.17 |  | -1.51 |  | -1.48 |  |
|  | *2* | *5038* | 0.01 |  | 0.01 |  | 0.01 |  | 0.01 |  | 5.50 |  | 3.86 |  | 4.50 |  |

****p<0.001 **p<0.01 *p<.05* The numbers next to the variables are the Average Treatment on the Treated (ATT), below are T statistics. The models were estimated using Propensity Score Matching (PSM) with the nearest-neighbour and five-nearest-neighbours’ approaches.

**Table A4. Other ICDS assistance – in pregnancy and with breastfeeding – and children’s nutrition outcomes**

|  | *N untreated off support* | *N untreated on support* | **Stunted** | | **Underweight** | | **Wasted** | | **Anaemic** | | **ZHA** | | **ZWA** | | **ZWH** | |
| --- | --- | --- | --- | --- | --- | --- | --- | --- | --- | --- | --- | --- | --- | --- | --- | --- |
|  | *N treated off support* | *N treated on support* |  |  |  |  |  |  |  |  |  |  |  |  |  |  |
| **ICDS assistance in pregnancy** |  |  |  |  |  |  |  |  |  |  |  |  |  |  |  |  |
| **Pre-pandemic** |  |  |  |  |  |  |  |  |  |  |  |  |  |  |  |  |
| *Nearest neighbour* |  | *3346* | 0.02 |  | -0.01 |  | -0.02 |  | 0.01 |  | -6.69 |  | 2.86 |  | 9.47 |  |
|  | *299* | *17856* | 0.02 |  | 0.02 |  | 0.02 |  | 0.02 |  | 8.22 |  | 5.27 |  | 6.54 |  |
| *Five nearest neighbours* |  | *3346* | 0.03 |  | -0.02 |  | -0.02 |  | 0.03 |  | -8.55 |  | 2.17 |  | 9.73 |  |
|  | *299* | *17856* | 0.02 |  | 0.02 |  | 0.01 |  | 0.01 |  | 7.09 |  | 4.55 |  | 5.69 |  |
| **Pandemic** |  |  |  |  |  |  |  |  |  |  |  |  |  |  |  |  |
| *Nearest neighbour* |  | *1000* | 0.01 |  | 0.03 |  | 0.00 |  | 0.01 |  | -14.32 |  | -12.69 |  | -9.66 |  |
|  | *643* | *9295* | 0.04 |  | 0.03 |  | 0.03 |  | 0.04 |  | 15.68 |  | 10.49 |  | 12.53 |  |
| *Five nearest neighbours* |  | *1000* | 0.02 |  | 0.06 |  | 0.04 |  | 0.01 |  | -10.84 |  | -16.13 |  | -16.44 |  |
|  | *643* | *9295* | 0.03 |  | 0.03 |  | 0.03 |  | 0.03 |  | 13.47 |  | 9.07 |  | 10.75 |  |
| **ICDS assistance with breastfeeding** |  |  |  |  |  |  |  |  |  |  |  |  |  |  |  |  |
| **Pre-pandemic** |  |  |  |  |  |  |  |  |  |  |  |  |  |  |  |  |
| *Nearest neighbour* |  | *4354* | 0.02 |  | -0.01 |  | 0.02 |  | 0.01 |  | 0.72 |  | -4.21 |  | -6.26 |  |
|  | *8* | *17139* | 0.02 |  | 0.02 |  | 0.02 |  | 0.02 |  | 7.71 |  | 5.04 |  | 6.28 |  |
| *Five nearest neighbours* |  | *4354* | 0.03 |  | -0.01 |  | 0.00 |  | 0.01 |  | -7.46 |  | -6.28 |  | -3.05 |  |
|  | *8* | *17139* | 0.02 |  | 0.02 |  | 0.01 |  | 0.01 |  | 6.70 |  | 4.37 |  | 5.44 |  |
| **Pandemic** |  |  |  |  |  |  |  |  |  |  |  |  |  |  |  |  |
| *Nearest neighbour* |  | *1230* | -0.02 |  | -0.04 |  | -0.01 |  | 0.00 |  | 4.56 |  | 2.07 |  | -2.73 |  |
|  | *392* | *9316* | 0.04 |  | 0.04 |  | 0.03 |  | 0.04 |  | 15.51 |  | 10.97 |  | 12.80 |  |
| *Five nearest neighbours* |  | *1230* | -0.02 |  | 0.00 |  | 0.01 |  | -0.01 |  | 5.59 |  | 2.48 |  | -1.09 |  |
|  | *392* | *9316* | 0.03 |  | 0.03 |  | 0.03 |  | 0.03 |  | 13.62 |  | 9.54 |  | 11.04 |  |

****p<0.001 **p<0.01 *p<.05* The numbers next to the variables are the Average Treatment on the Treated (ATT), below are T statistics. The models were estimated using Propensity Score Matching (PSM) with the nearest-neighbour and five-nearest-neighbours’ approaches.
